# De novo transcriptomic analysis and identification of EST-SSR markers in *Stephanandra incisa*

**DOI:** 10.1038/s41598-020-80329-7

**Published:** 2021-01-13

**Authors:** Cuiping Zhang, Zhonglan Wu, Xinqiang Jiang, Wei Li, Yizeng Lu, Kuiling Wang

**Affiliations:** 1 College of Landscape Architecture and Forestry, Qingdao Agricultural University, Qingdao, 266109 China; 2 Shandong Provincial Center of Forest Tree Germplasm Resources, Jinan, 250102 Shandong China

**Keywords:** Genetics, Molecular biology, Plant sciences

## Abstract

*Stephanandra incisa* is a wild-type shrub with beautiful leaves and white flowers and is commonly used as a garden decoration accessory. However, the limited availability of genomic data of *S. incisa* has restricted its breeding process. Here, we identified EST-SSR markers using de novo transcriptome sequencing. In this study, a transcriptome database containing 35,251 unigenes, having an average length of 985 bp, was obtained from *S. incisa*. From these unigene sequences, we identified 5,555 EST-SSRs, with a distribution density of one SSR per 1.60 kb. Dinucleotides (52.96%) were the most detected SSRs, followed by trinucleotides (34.64%). From the EST-SSR loci, we randomly selected 100 sites for designing primer and used the DNA of 60 samples to verify the polymorphism. The average value of the effective number of alleles (*Ne*), Shannon’s information index (*I*), and expective heterozygosity (*He*) was 1.969, 0.728, and 0.434, respectively. The polymorphism information content (*PIC*) value was in the range of 0.108 to 0.669, averaging 0.406, which represented a middle polymorphism level. Cluster analysis of *S. incisa* were also performed based on the obtained EST-SSR data in our work. As shown by structure analysis, 60 individuals could be classified into two groups. Thus, the identification of these novel EST-SSR markers provided valuable sequence information for analyzing the population structure, genetic diversity, and genetic resource assessment of *S. incisa* and other related species*.*

## Introduction

*Stephanandra incisa* is a wild-type shrub belonging to the genus *Stephanandra* and is widely distributed in eastern Asia^1^. Due to its peculiar leaf shape, and white flowers in clusters, it is commonly used as an ornament for garden decoration. Additionally, its wood is expensive and is used for construction purposes^2,3^. Studies on *S. incisa* have primarily focused on its distribution, system evolution, cultivation, and breeding^4,5^. However, limited knowledge on the molecular markers of *S. incisa*, especially codominant markers have restricted further research efforts to understand the genetic and molecular biological processes of this species.

Molecular markers are efficient tools that facilitate genetic diversity analysis and selection of the germplasm^6,7^. One of the most common molecular markers include simple sequence repeats (SSR) due to their polymorphism, high stability, cross-transferable nature, and simplicity of analysis^8,9^. They are randomly distributed across the genome in several plant species. The SSRs are generally classified into expressed sequence tag-SSRs (EST-SSRs) and genomic-SSRs (gSSRs) based on the original sequences. EST-SSRs are more efficient as well as time-and cost-effective compared with g-SSRs^10^. Additionally, EST-SSRs are derived from mRNA transcript sequences, are more evolved, and exhibit more transferability between species^11,12^. Until now, several EST-SSRs have been identified, and used in breeding system research, construction of genetic linkage map, and genetic diversity analysis to evaluate EST-SSRs polymorphism in various plant species^13–15^. However, there are no published reports on the identification of EST-SSRs in *S. incisa*.

High throughput RNA-seq is a next-generation sequencing (NGS) technology, which provides large-scale genomic and transcriptomic information in the fields of conservation genetics and evolution^16,17^. NGS technology is also used for generating genome-based, functional data for non-model plants, such as *Rhododendron latoucheae*, and numerous EST sequences for gene annotation and target discovery analysis as well as comparative genomics^18,19^. Previously, genomic analysis was done using conventional sequencing methods; however, it had several disadvantages, such as high cost, labor-intensive, and time-consuming. The newly developed NGS technology, especially de novo transcriptome sequencing, is capable of generating large volumes of information to be used for detecting molecular markers, novel gene discovery, and identification of polymorphic loci^20,21^. This technique can be used for the analysis of model as well as non-model plants^22^. The high throughput and cost-efficient features highlight the accuracy and efficiency of the de novo transcriptome sequencing, which has made it the method of choice in life sciences research^23,24^. It has used to identify several EST sequences in several plants, such as *Pinus bungeana*, *Lemna gibba*, *Curcuma alismatifolia*, *Styrax japonicus*, and *Sophora japonica*^6,14,25–27^.

Here, the Illumina sequencing platform was used to construct the first transcriptome of *S. incisa* to (1) assemble and annotate the transcriptome data of *S. incisa*, (2) develop and identify several EST-SSRs markers for *S. incisa*, and (3) analyze polymorphism of these EST-SSRs markers among Laoshan (LS) and Anshan (AS) populations to further develop and utilize *S. incisa* EST resources and (4) analyze genetic identity based on the obtained EST-SSR data. This information will contribute to further the genetic diversity research and assessment of *S. incisa*.

## Results

### Illumina sequencing and de novo assembly

**Figure 1:**
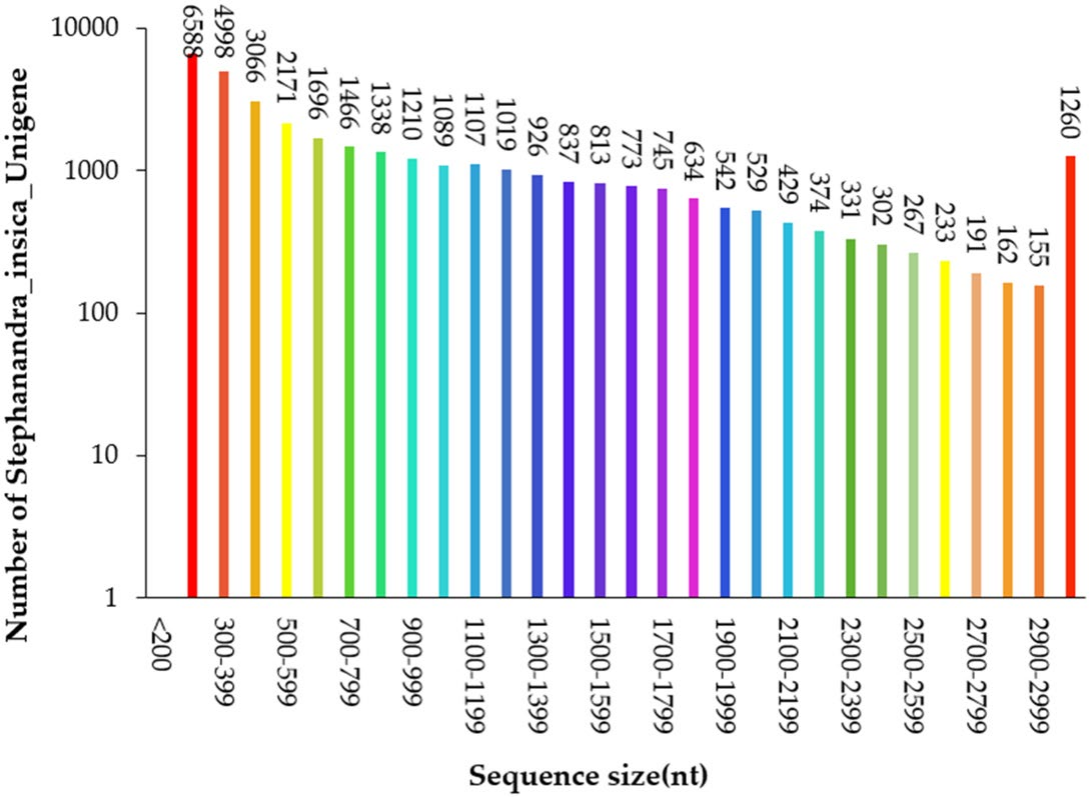
Sequence length distribution of the assembled unigenes.

Of the 40,053,100 raw reads generated for *S. incisa*, 40,010,736 clean reads containing 97.98% Q20 bases were generated post strict filtration and quality control. The reads contained 5,968,100,025 nucleotides (nt), with 48.72% GC content. We identified 35,251 unigenes having an average length of 985 bp and with an N50 and N60 value of 7,212 and 23,437 bp, respectively. The unigenes were 201 to 11,634 bp long with a total length of 34,743,756 bp (Table 1). In the final unigene collection, 14,652 unigenes (41.56%) were 200 to 499 bp long, 7,881 unigenes (22.36%) were 500 to 999 bp long, 4978 unigenes (14.12%) were 1000 to 1499 bp long, 3507 unigenes (9.95%) were 1500 to 1999 bp long, and 4233 unigenes (12.01%) were longer than 2000 bp. The results showed that with an increase in the length of the unigenes sequence, there was a decrease in the number of assembled unigenes (Fig. 1).

**Table 1:** De novo assembled EST-SSRs for *S. incisa.*

Category	Items	Numbers
Raw reads	Total raw reads	40,053,100
Clean reads	Total clean reads	40,010,736
	Total clean nucleotide (nt)	5,968,100,025
	Q20 percentage	97.98%
	N percentage	0.00%
	GC percentage	48.72%
Unigenes	Total sequence number	35,251
	Total sequence base	34,743,756
	Largest	11,634
	Smallest	201
	Average	985
	N50 (bp)	7212
	N90 (bp)	23,437
EST-SSRs	Sequences examined	35,251
	Size of examined sequences (bp)	34,743,756
	Identified SSR	5555
	SSR-containing sequences	4519
	Sequences containing more than one SSR	846
	SSR present in compound formation	495

### Functional annotation and classification

**Figure 2:**
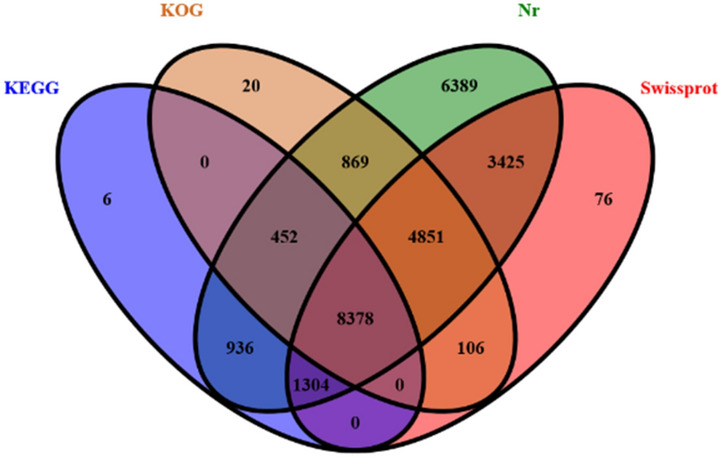
Venn diagram of annotation.

We compared the unigenes obtained by transcriptome sequencing with the Swiss-Prot, Nr, KOG, and KEGG databases (Fig. 2). A total of 35,251 unigenes were annotated. The results showed that 26,604 (Nr: 75.47%), 18,140 (Swissprot: 51.46%), 14,676 (KOG: 41.63%), and 11,067 (KEGG: 31.42%) unigenes were functionally annotated. Among these, 8830 (25.42%) unigenes were annotated in both the KEGG and the KOG databases. However, only 2246 (6.37%) were annotated in the KEGG database. Additionally, 14,550 (41.28%) unigenes were annotated in both the KOG and the Nr databases (Fig. 2).

The results of the comparative analysis of the homology of the *S. incisa* transcriptome unigenes in the Nr database found highest homology with *Prunus mume* unigene. We found that 11,565 unigenes were annotated to *Prunus mume*, 3561 unigenes to *Malus domestica*, and 3189 unigenes to *Pyrus x bretschneideri*. It exhibited low homology with *Medicago truncatula* and *Arabidopsis thaliana* (Figure S1 and Table S1).

**Figure 3:**
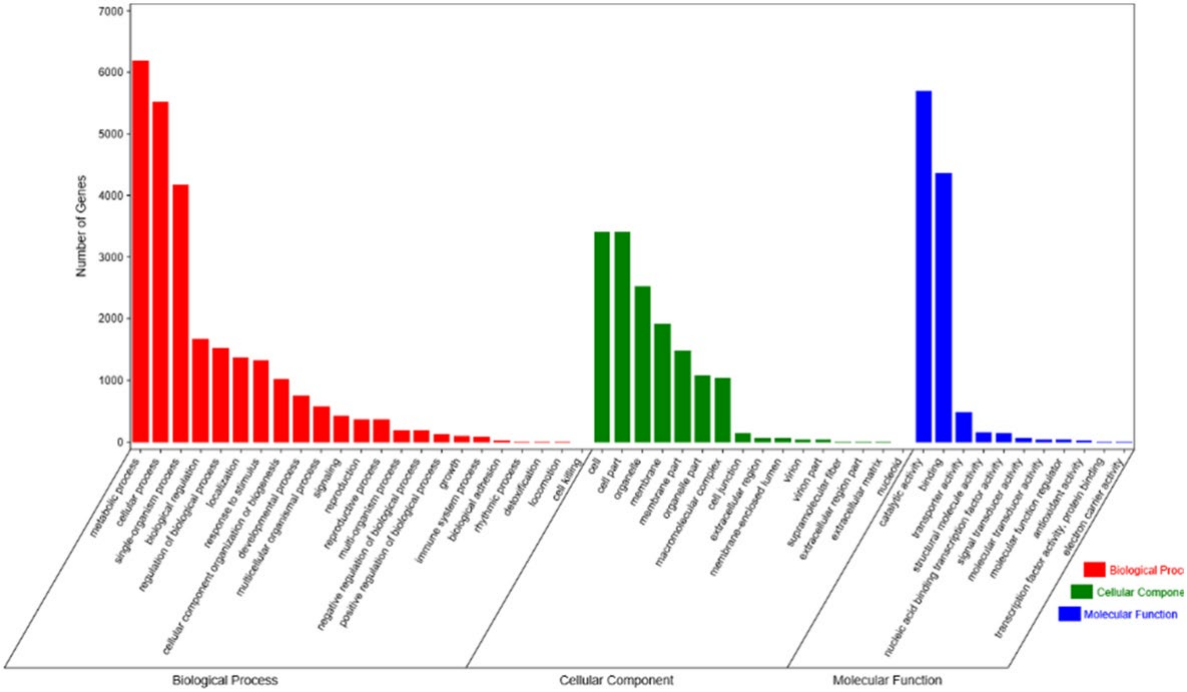
Gene Ontology (GO) classification of *Stephanandra incisa* unigenes (the y-axis indicates the number of unigenes in the GO databases. The y-axis indicates Gene Ontology (GO) classification of *Stephanandra incisa* unigenes).

GO annotated unigenes were classified into the following three main categories: molecular function, cellular component, and biological processes. Unigenes in these three biological function categories included 50 level-2 categories. Amongst the biological processes, the largest group was comprised of unigenes of metabolic process (6195; 11.83%), followed by cellular process (5,517; 10.54%). Amongst the cellular components, the largest group was comprised of unigenes of cell (3401; 6.50%) and cell parts (3401; 6.50%), followed by organelle (2531; 4.83%) and membranes (1918; 3.66%). Amongst the molecular function, the largest group was comprised of unigenes of catalytic activity (10.90%, 5706), followed by binding (4362; 8.33%) (Fig. 3 and Table S2).

**Figure 4:**
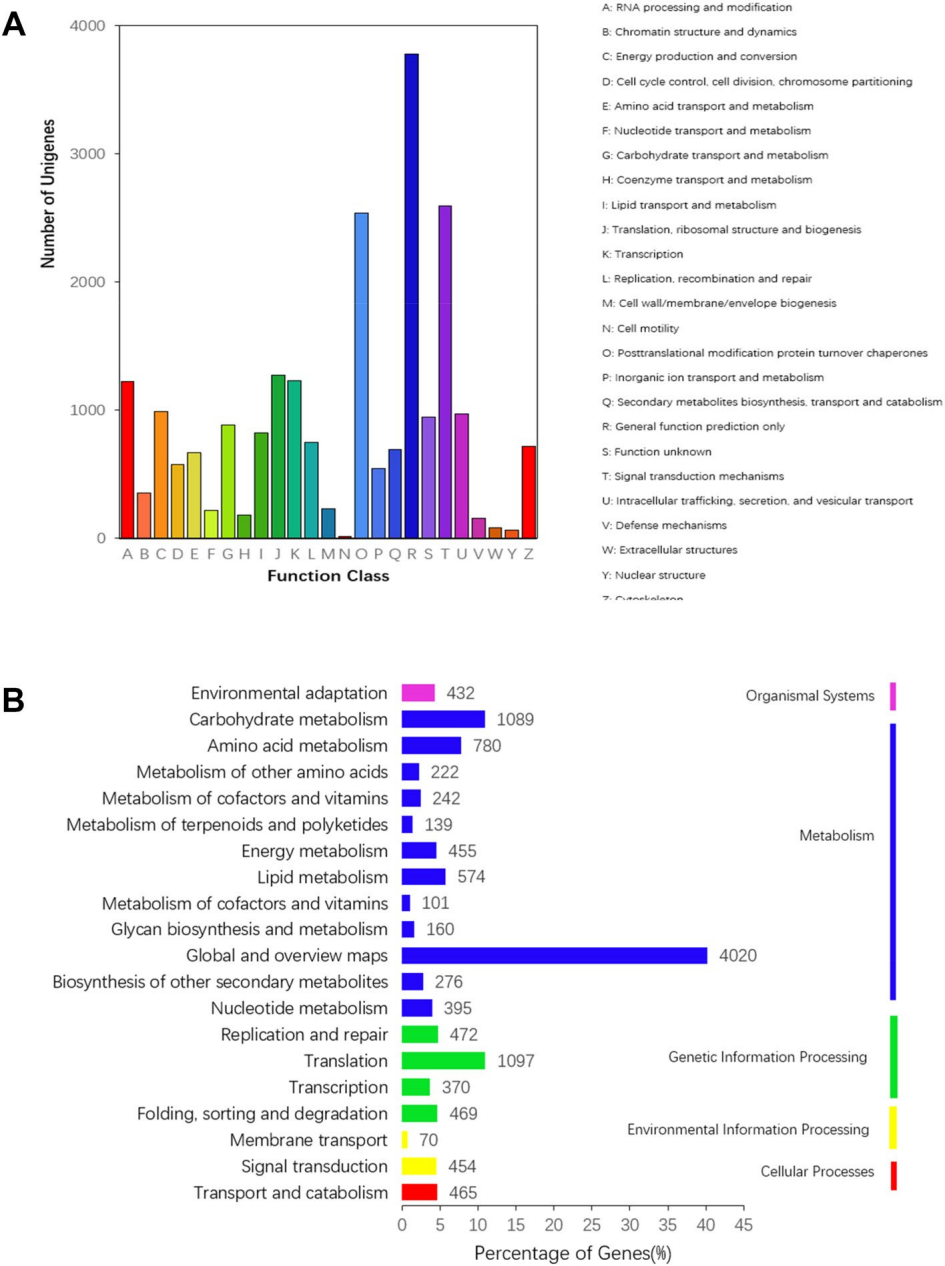
** (A)** KOG functional classification of *S. incisa* unigenes (The y-axis indicates the number of unigenes in a specific functional cluster. The x-axis indicates the KOG functional classification). (**B)** KEGG metabolic pathway of *S. incisa* (the y-axis is the name of the KEGG metabolic pathway, and the x-axis is the ratio of the number of genes. https://www.genome.jp/dbget-bin/www_bget?pathway+map01100).

For the KOG classification, 14,676 (41.63%) annotated unigenes were classified into 25 molecular families. The dominant group was comprised of general function prediction only (3775; 16.79%), followed by signal transduction mechanisms (2,593; 11.53%), protein turnover, post-translational modification, chaperones (2540; 11.29%), and ribosomal structure, translation, and biogenesis (1274; 5.66%). The smallest group was comprised of cell motility (17; 0.076%). Other classifications were more average in proportion, mostly containing approximately 1000 unigenes (Fig. 4A and Table S3).

Next, 11,067 unigenes were classified into five categories, including 133 KEGG pathways. The largest category was comprised of metabolism (8453; 76.38%), followed by the genetic information processing (2408; 21.76%), environmental information processing (524, 4.73%), cellular processes (465, 4.20%), and organismal systems (432, 3.90%) (Fig. 4B and Table S3).

### Frequency and distribution of SSRs in the unigenes

**Figure 5:**
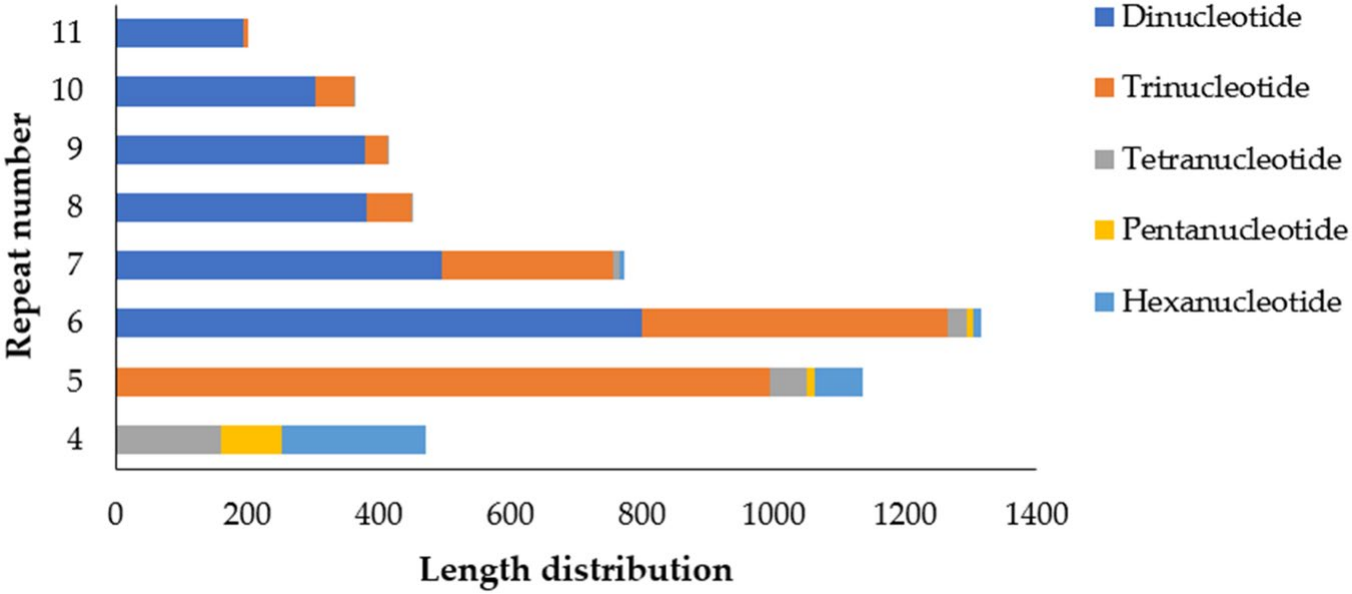
Length distribution of the EST-SSRs of *S. incisa* based on the number of nucleotides repeat units.

Of the 4519 unigenes, we identified 5555 potential EST-SSRs, amongst which 846 unigenes had greater than one EST-SSR locus, and 495 were identified as compound microsatellites (Table 1). The distribution density of SSRs in the *S. incisa* unigenes was one SSR per 1.60 kb (5555 SSRs in 34.74 Mb) at a frequency of 41.14%. The most common SSRs were di-nucleotide SSRs (2,942, 52.96%), followed by tri-nucleotide (1924, 34.64%), and hexa-nucleotide (313, 5.63%). The remaining SSRs accounted for 6.76% of the total SSRs. Additionally, the most common repeats of the EST-SSR were six tandem repeats (1317, 23.71%), followed by five tandem repeats (1137, 20.47%), seven tandem repeats (774, 13.93%), four tandem repeats (475, 8.55%), eight tandem repeats (454, 8.17%), nine tandem repeats (415, 7.47%), ten tandem repeats (365, 6.57%). The remaining tandem repeats accounted for < 12% of the EST-SSR (Fig. 5 and Table S4).

**Figure 6:**
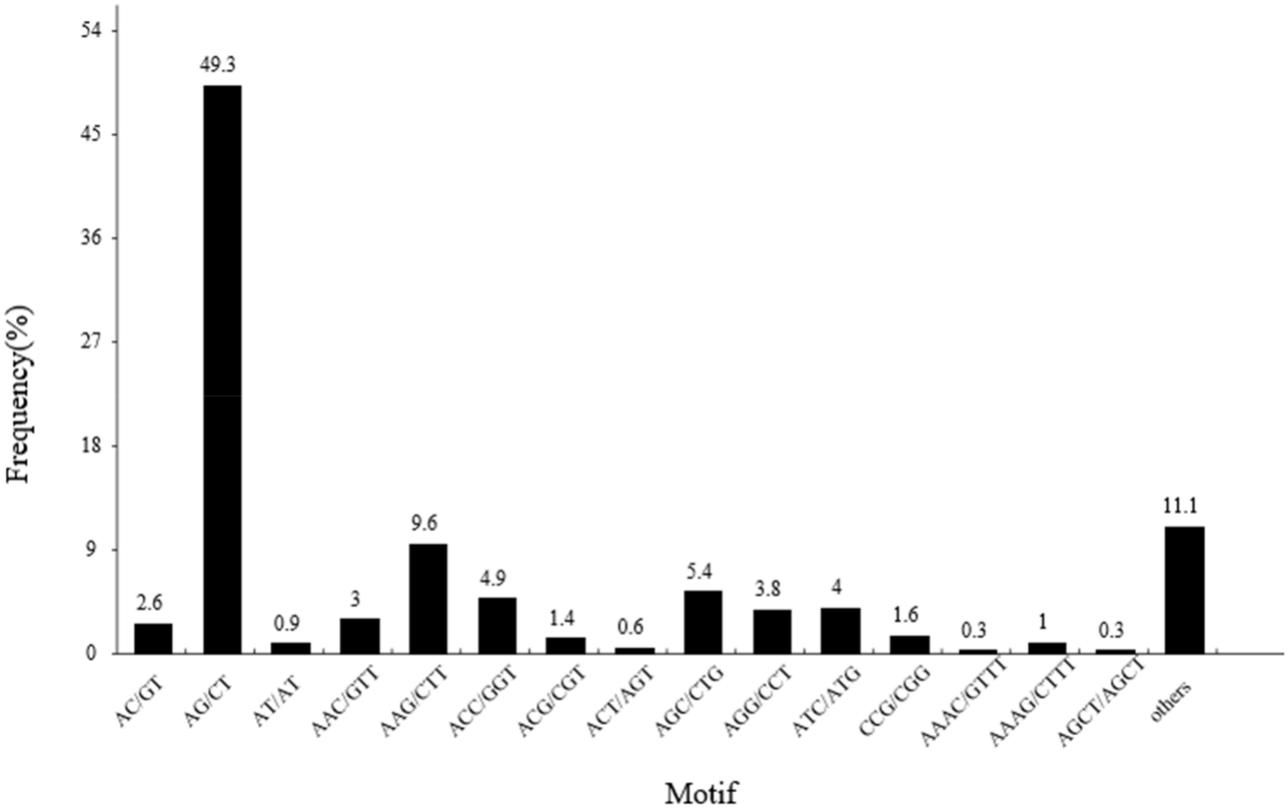
Distribution of SSR Motifs.

We observed a significant motif-type bias in *S. incisa* (Fig. 6). The most common motif in the di-nucleotide repeats was AG/CT (2739, 49.31%), followed by AC/GT (147, 2.65%), and AT/AT (51, 0.92%). The most common motif in the tri-nucleotide repeats was AAG/CTT, followed by AGC/CTG (300, 5.40%), ACC/GGT (270, 4.86%), ATC/ATG (224, 4.03%), AGG/CCT (213, 3.83%), AAC/GTT (169, 3.04%), CCG/CGG (90, 1.62%), ACG/CGT (79, 1.42%), and ACT/AGT (33, 0.59%). These 15 repeat motifs accounted for 88.91% of the total motifs, while the remaining types accounted for 11.09% (Fig. 6 and Table S5).

### Development and validation of novel EST-SSRs

**Figure 7:**
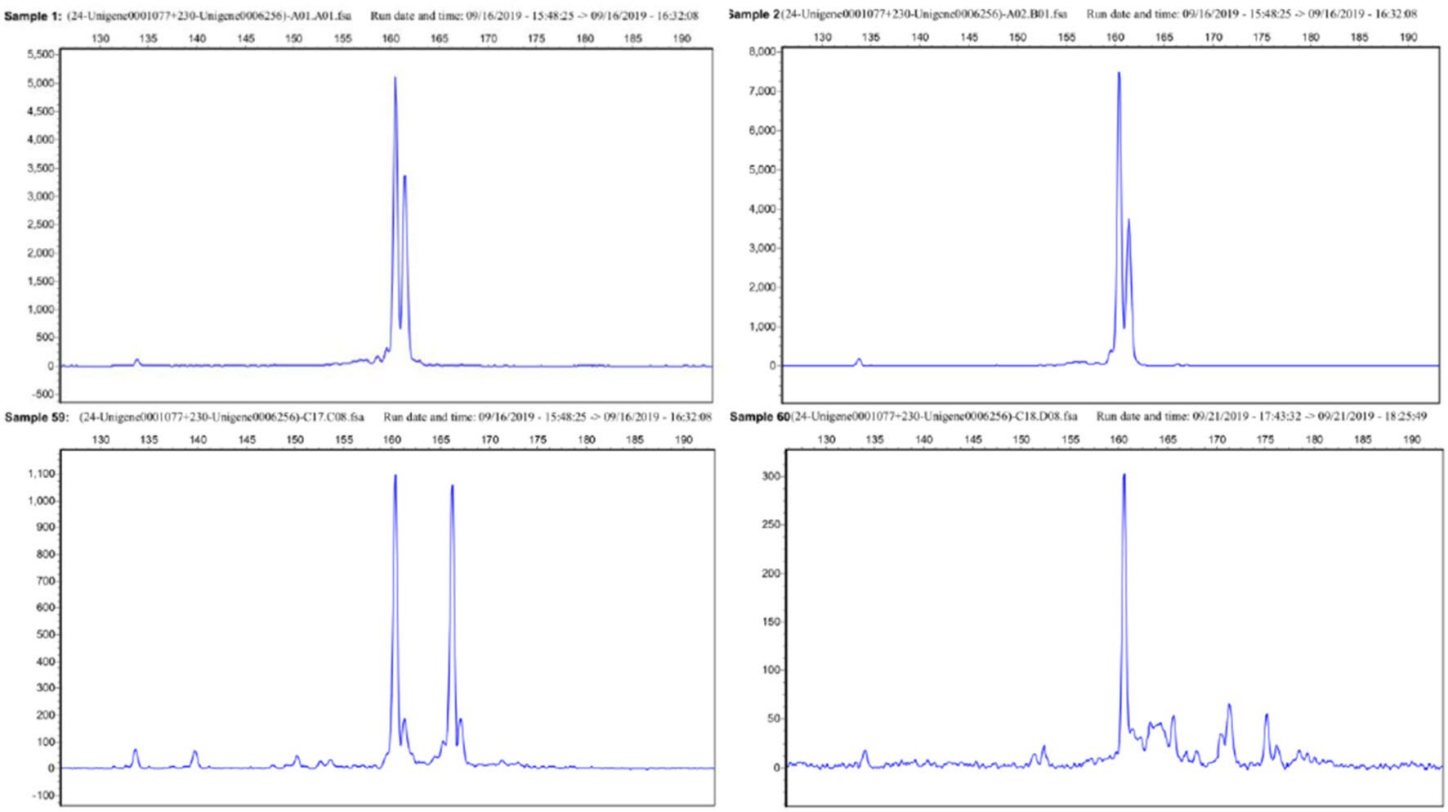
Partial capillary electrophoresis diagram of 60 samples (Genemarker2.2.0; https://genemarker.software.informer.com/2.2/).

We used Primer5 software to design primer pairs that meet the required criteria. We randomly selected and synthesized 100 primer pairs, and initially tested the polymorphism of SSR primers. 29 primer pairs with polymorphism were separated by agarose gel (Figure S2). Finally, amplicons and amplicon polymorphisms were assessed using DNA from 60 samples. The polymorphisms of loci were detected by capillary electrophoresis (Fig. 7).

### Polymorphism of SSR loci

The results showed that there were 29 polymorphic loci in 100 loci. Additionally, we detected 90 alleles in these two populations, at an average of 3.1 alleles/locus. S21 was the locus with the largest number of alleles (6), with the values for *Ne*, *He*, and *PIC* as 2.933, 0.665, and 0.592, respectively. The value of *Ne* lied in the range of 1.130 and 3.569 with an average of 1.969. S8 has the highest value of *I* (1.324) and S3 had the lowest value of *I* (0.230) with an average value of 0.728. The value of *He* ranged from 0.070 to 0.723 with a mean of 0.434. The value of *PIC* varied from 0.108 to 0.669 with an average of 0.406. For overall *PIC*, nine loci had values > 0.50 (highly informative), while S01, S03, S04, S06, S13, and S19 had values < 0.25 (minimally informative), and the remaining 14 loci had values between 0.25 and 0.50 (moderately informative; Table S6).

### Cluster analysis

All the amplified alleles were used for cluster analysis of the 60 individuals via the UPGMA method. A UPGMA dendrogram showed that all the individuals were divided into two clusters, in which cluster 1 contained A27, LS06, LS07, LS23 and cluster 2 contained all other individuals (Figure S3). Clearly, a high level of genetic similarity was displayed, with similarity coefficients distributed in a narrow range of 0.75–1.0. The results indicated low diversity in the screened germplasm and narrow genetic.

## Discussion

SSR markers are commonly used for genetic diversity analysis, constructing cultivar DNA fingerprinting, marking assisted breeding, and studying genome-wide association^28^. Transcriptome sequencing has been efficiently used to develop SSR markers in several plant species. It can also be used in non-model plants and plays a crucial role in the discovery of novel genes, gene expression analysis, and for the identification of molecular markers^29–31^. *S. incisa* is a non-model plant with high ornamental and medicinal value. However, there are no published reports on the transcriptome sequence of *S. incisa*. In this study, the transcriptome of *S. incisa* was reported using Illumina sequencing technology. We identified 40,053,100 paired-end raw reads and 40,010,736 high-quality clean reads with a Q20 level of 97.98%, to ensure the quality of sequencing. The assembled unigenes were shorter than *Saccharina japonica* (44,362,190) but longer than Chinese Hawthorn (28,888,844)^32,33^. The average length of these unigenes was longer than that of *Pinus bungeana* (922 bp) and *Dalbergia odorifera* (676 bp)^27,34^. Next, these assembled unigenes were successfully annotated to registered public databases, including Swiss-Prot, Nr, KEGG, and KOG. These annotations provide useful information, which could be used for the genetic diversity analysis of *S. incisa* in the future. The result of the annotation showed that *S. incisa* was closely related to *Prunus*, *Housefly*, and *Pyrus x bretschneideri*, all belonging to the family *Rosaceae*. These results were consistent with the previous taxonomic studies. In the GO classification results, metabolic processes and catalytic activity were classified as the largest groups among the three functional categories. The prediction cluster occupied an important position in the KOG classification, consistent with previous results, followed by signal transduction mechanisms and protein turnover, posttranslational modification, chaperones categories. As for KEGG pathways database, 11,076 (31.42%) unigenes had high matches and were classified to five categories, including 133 KEGG pathways. Thus, these results provided valuable information to be used to study genetic diversity and improved adaptation of *S. incisa*.

Molecular markers can provide adequate genetic diversity information for plants^35–37^. EST-SSR markers have been an effective tool for analyzing gene structure, linkage mapping, and QLT analysis^38^. Thus, the identification of EST-SSR markers of *S. incisa* could promote its molecular breeding process, especially in mapping and anchoring parental maps. However, there exist no published reports on the molecular markers in *S. incisa*. Here, we identified 5555 EST-SSRs, among which, dinucleotide repeat sequences and trinucleotide repeat sequences accounted for a large proportion. The most common dinucleotide repeat in *S. incisa* was AG/CT, followed by AC/GT, and AT/AT. Although the functional significance of SSRs in plant transcript regions is unclear, the AG/CT motif, a homopurine-homopyrimidine stretch frequently found in the 5′ untranslated region, reportedly plays a vital role in regulating the gene expression and nucleic acid metabolism in plants^39^. The most common trinucleotide repeat was AAG/CTT, followed by AGC/CTG, and ACC/GGT. These results agreed with the results of previous research and showed that the AAG may be the most important motif in dicotyledonous plants^40^. Additionally, the results agreed with the previous studies on the *Rosacea*, such as *Prunus mume*^41^. Thus, the frequency of SSRs was closely linked to the size of the database, mining tool used, the genome structure, and the species differences^42^.

The location site of EST-SSR markers in the genes facilitates their use for detecting valuable genetic diversity that is possibly associated with valuable breeding traits. The EST-SSRs are considered appropriate for designing specific primers due to the high quality amplicons^43^. In this study, we randomly selected and synthesized 100 primer pairs, followed by the identification of 29 polymorphic genic SSR markers and 90 alleles among the two *S. incisa* populations. Additionally, the average values of *Ne*, *I*, and *He* were 1.969, 0.728, and 0.434, respectively. These results indicated that *S. incisa* has a medium level of genetic diversity. The *PIC* value is used to assess the level of genetic information^44^. Additionally, it is used employed to assess the polymorphic level of the markers and classified into high-level (< 0.5), moderate-level (0.5 < *PIC* < 0.25), and low-level (< 0.25) categories^45^. The value of *PIC* ranged from 0.108 to 0.669, which represented a middle polymorphism level. The level of polymorphism could be attributed to several factors, such as the difference in quantity in the sample. Here, polymorphism was detected based on the identified EST-SSR-containing sequences, which might be a valuable resource for the identification of genetic markers for future research on *S. incisa*. An admixture model-based approach was implemented to evaluate the population structure, and suggested two clusters were the best for the 60 *S. incisa* samples. The analysis of neighbor-joining confirmed this result. Based on the cluster analysis results, no obvious correlation of genotypes of the *S. incisa* to their geographical locations.

These polymorphic microsatellite markers would provide an important reference for developing polymorphic molecular markers for *S. incisa* and could be applied in population genetics analysis, linkage mapping, and marker-assisted selective breeding in the future. Thus, the identified EST-SSR makers were effective for the genetic analysis of the *S. incisa* populations. Additionally, these results could act as a new valuable resource for genomic studies on *S. incisa*. However, only 60 individuals from two groups were genetically analyzed. Thus, further research with an extended sample size needs to be conducted for the analysis of genetic diversity and structure of the species.

## Conclusions

This is the first study to analyze the transcriptome of *S. incisa*. A total of 35,251 unigenes, with an average length of 985 bp, were obtained from *S. incisa* to create a transcriptome database, from which 5555 EST-SSRs were identified. All unigenes were annotated in the Nr (75.47%), Swiss-Prot (51.46%), KOG (41.63%), and KEGG (31.42%) databases. From these EST-SSR loci, we randomly selected 100 sites for designing primer and used the DNA of 60 samples to verify the polymorphism. Diversity parameters showed that these primer pairs had a middle polymorphism level among the two *S. incisa* populations. Cluster analysis indicated that 60 individuals could be classified into two groups. This is the first report to identify EST-SSR markers in *S. incisa*, which could be used for further genetic research and breeding approaches in *S. incisa*.

## Methods

### Plant materials and RNA isolation

**Figure 8:**
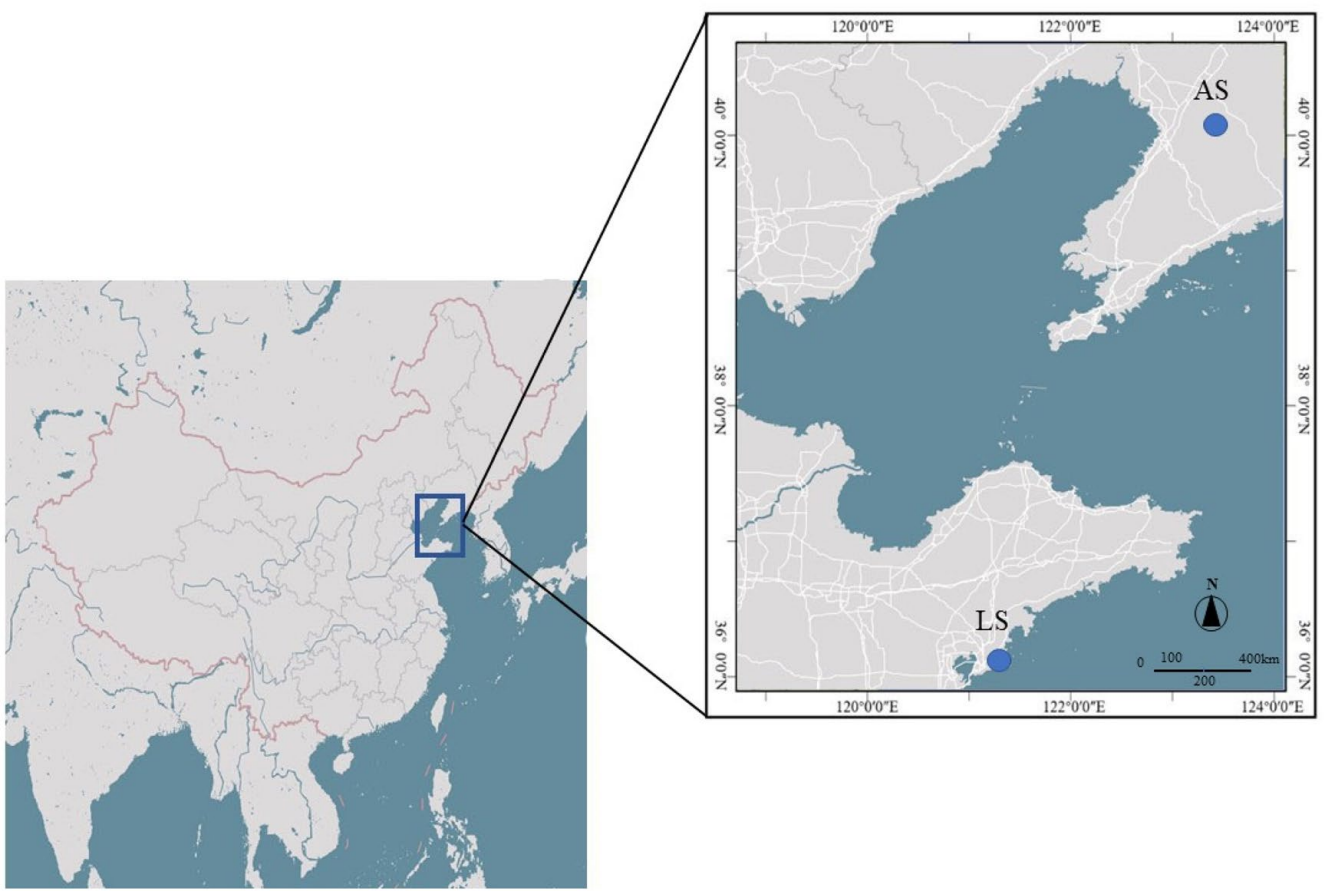
Geographic distribution of the two investigated *S. incisa* populations sampled from China. ArcMap10.5 (http://www.esri.com/) was used to generate the map.

We collected grown leaves of *S. incisa* plants from Laoshan to conduct transcriptomics analysis. The leaves were flash frozen in liquid nitrogen and stored at − 80 °C until further use. The TRIzol reagent was used for extracting total RNA following the specified protocol (Invitrogen, USA). For confirming polymorphism, 60 individual samples from the Laoshan (LS) and Anshan (AS) populations were selected, and each of the sampled individuals was kept more than 150 m apart to minimize the genetic relationships among the sample trees (Fig. 8). The fresh and healthy leaves were stored in silica gel. The total genomic DNAs were extracted using CTAB^46^. The extracted RNA and DNA were quantified using Thermo Scientific Nanodrop2000 spectrophotometer. Agarose gel electrophoresis was used to assess the integrity of the quantified DNA and RNA. The high-quality RNA samples were used to prepare the complementary cDNA library.

### Construction of cDNA library and illumina sequencing

Following the Illumina sequencing platform manufacturer’s instructions, first, the first and the second strand were synthesized. Next, the cDNA was purified, ends were repaired, ligated to the adapter, followed by library enrichment. The cDNA quality and concentration were evaluated using Qseq100 DNA Analyzer (Bioptic Inc, China). Finally, the Illumina HiSeq 2000 platform was used to sequence the cDNA library.

### Sequence assembly and data analysis

The raw sequencing data can be filtered by removing the adapter contaminants, reads containing a high number of poly-N, and read data with < 20 bp. The SeqPrep program can be used to screen high-quality clean read data for the de novo assembly, empty reads, and reads with Q < 20, followed by the calculation of the GC content, sequence replication levels, along with the Q20 and Q30 values of the obtained clean data^47^. Thus, we filtered the raw sequencing reads (reads with unknown base ‘N’, low-quality reads with the adapter, along with other low-quality sequences) to obtain the clean reads, which were assembled using Trinity software (version 2.4.0, https://github.com/trinityrnaseq/trinityrnaseq/issues/270) to get the consensus sequences. The software combined the reads with overlapping nucleic acid sequences into contigs. The sequences that could not extend at both ends of contigs were classified as unigenes^48^. These assembled unigenes were used for annotation analysis. These unigenes were compared with public databases (*E* < 1e−5), such as NCBI non-redundant (Nr, http://www.ncbi.nlm.nih.gov/)^49^, Swiss-Prot (http://www.expasy.ch/sprot/)^50^, KOG (http://www.ncbi.nlm.nih.gov/COG), and KEGG (http://www.genome.jp/kegg/) via BLASTX (version 2.2.26, ftp://ftp.ncbi.nlm.nih.gov/blast/executables/blast+/LATEST/) to get information on classification and functional annotation^51^. From the NCBI Nr database, Blast2GO was used to attain the gene ontology (GO) annotation information of unigenes to identify their molecular functions, cellular components, and biological processes^52^. For KEGG pathway analysis, BLASTX software was used to perform the required operations to construct KEGG database^53^.

### Identification of EST-SSRs loci and primer design

The SSR loci contain di/tri/tetra/penta/hexa nucleotide sequences with at least 8/6/4/3/3 repeats, respectively. The Microsatellite identification tool (MISA, http://www.gramene.org/db/markers/ssrtool) was used to identify EST-SSRs^54^. Primer 5.0 (http://www.premierbiosoft.com/primerdesign/) was used to design the primers based on the following parameters: annealing temperature (55–60 °C), GC content (40–60%), primer lengths (18–22 bp), and target PCR product size (100–300 bp)^55^. All primers were synthesized by the Beijing Ruibiotech Company.

### Validation and application of SSR markers

The CTAB method was used to extract total genomic DNA^56^. The PCR reactions were carried out in a final volume of 20 µL, which contained 2 × Mix (10 µL), reverse primer (0.15 µL), forward primer (0.15 µL), DNA (1 µL) and dd H2O up to 20 µL. PCR amplification was carried out at 95 ℃ for 5 min, followed by 35 denaturation cycles at 95 ℃ for 30 s, annealing at 52 ℃ for 30 s, and then extension at 72 ℃ for 30 s. A final extension at 72℃ and 10 min was also carried out. All the PCR products were verified through capillary electrophoresis. Electrophoretograms of capillary electrophoresis were read and analyzed through Gene Marker V2.2.0 (https://genemarker.software.informer.com/2.2/) software.

### Statistical analysis

The number of observed alleles (*Na*), expected heterozygosity (*He*), the number of effective alleles (*Ne*), and Shannon’s information index (*I*)^57^ were calculated using the POPGENE V1.32 (https://www.softpedia.com/get/Science-CAD/Popgene-Population-Genetic-Analysis.shtml) software. The Polymorphism information content (*PIC*) was calculated using the POWER-MARKER v3.25 (http://statgen.ncsu.edu/powermarker/index.html)^45^. Unweighted pair group (UPGMA) in NTSYS 2.1 (https://en.freedownloadmanager.org/Windows-PC/NTSYSpc.html) was used to cluster the populations of *S. incisa*, and a dendrogram was generated from this clustering.

## Supplementary Information

Supplementary Information

## Data Availability

The dataset is available from the NCBI Short Read Archive (SRA) with accession number SRR12158738.
