# Supplementary material for: De novo transcriptomic analysis and identification of EST-SSR markers in Stephanandra incisa
Source: Sci Rep. 2021 Jan 13;11:1059. doi: 10.1038/s41598-020-80329-7 (PMC7806653; doi:10.1038/s41598-020-80329-7)
Supplement: Supplementary file 1 — Supplementary Information [file 41598_2020_80329_MOESM1_ESM.pdf]

De novo transcriptomic analysis and identification of EST-SSR markers in *Stephanandra incisa*

Cuiping Zhang[1, +], Zhonglan Wu[1, +], Xinqiang Jiang[1], Wei Li[1], Yizeng Lu[2] & Kuiling Wang[1, *]

[1] College of Landscape Architecture and Forestry, Qingdao Agricultural University, Qingdao, 266109, China.

[2] Shandong Provincial Center of Forest Tree Germplasm Resources, Shandong Province, Jinan 250102, China.

*Corresponding author: klwang@qau.edu.cn

[+]These authors contributed equally to this work

Supplementary Figure 1

Statistics of annotation results for *Stephanandra incisa*

A: Corresponding annotation information

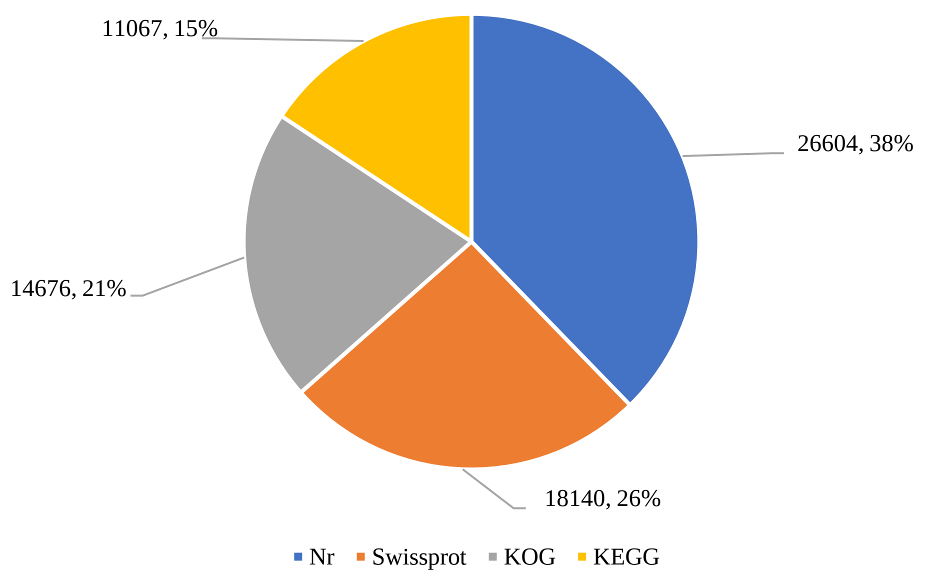

B: Species distribution classification map

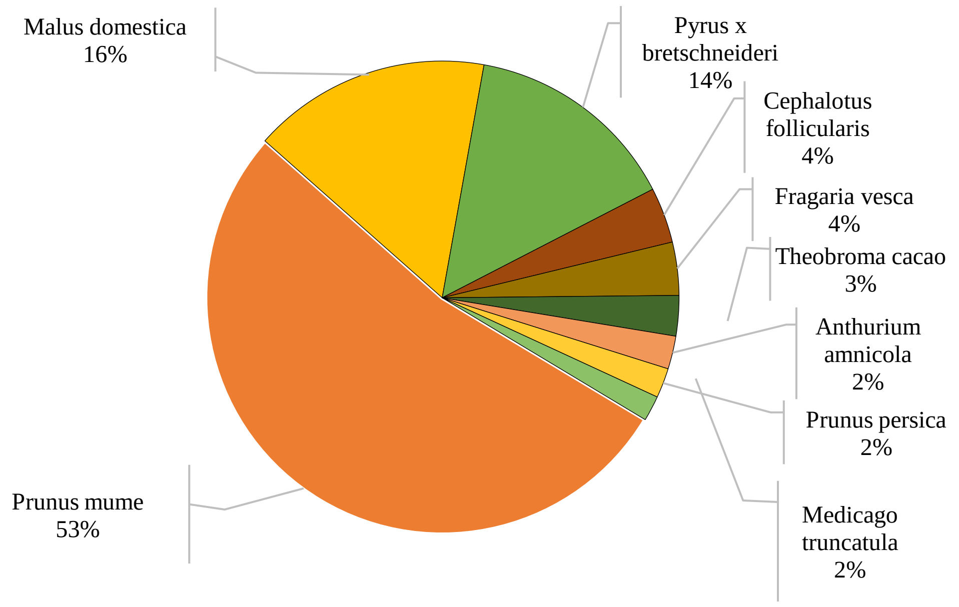

Supplementary Figure 2

Polymorphisms revealed by primer pair 29 of *S. incisa*

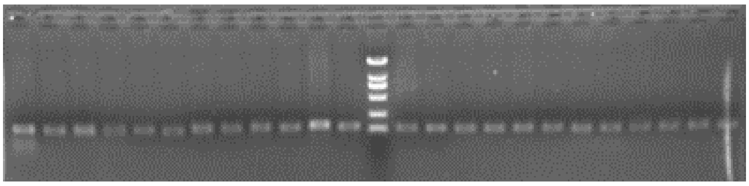

FORWARD PRIMER1 (5'-3'): CTCTCCTTCACACTAGCTCGG **REVERSE PRIMER1 (5'-3')**: AACATGGCCTCGTACACACA

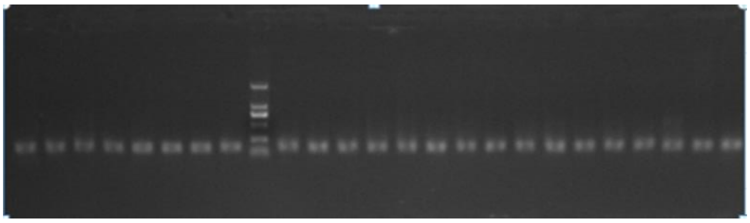

FORWARD PRIMER1 (5'-3'): GATAGAGCGCAAAGTGGAGG **REVERSE PRIMER1 (5'-3')**: TTATGGCTCTCTCTCCCCAA

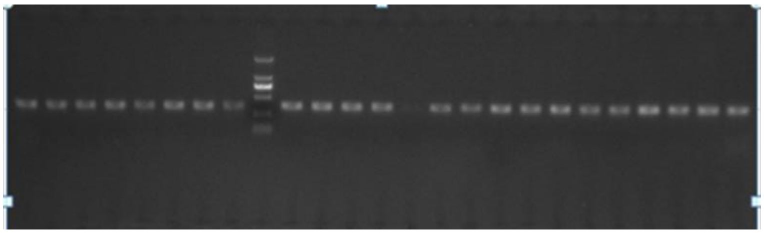

FORWARD PRIMER1 (5'-3'): TGATGACGTGCTTGTTCTCC **REVERSE PRIMER1 (5'-3')**: CACTCCCGGGAATCAAAAA

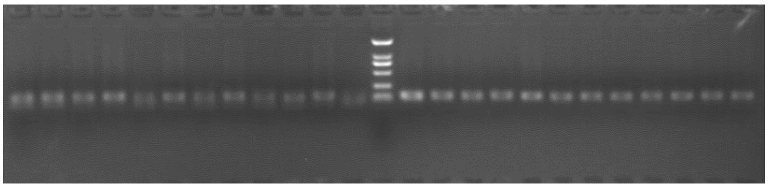

FORWARD PRIMER1 (5'-3'): GCACTCAGGAGGGAGTGAAG **REVERSE PRIMER1 (5'-3')**: GGACCTGGACTTGGAGTTGA

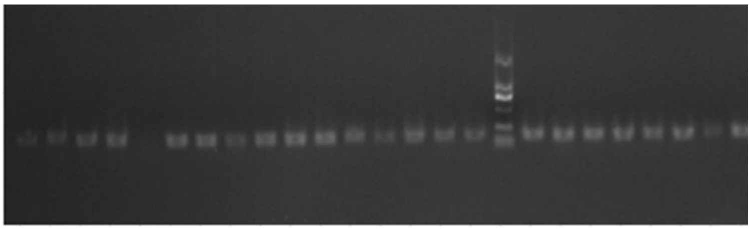

FORWARD PRIMER1 (5'-3'): GCGAGAAAATAGTGTTAGTGTGAGA **REVERSE PRIMER1 (5'-3')**: CCGCTTTTACCCTTTGATGA

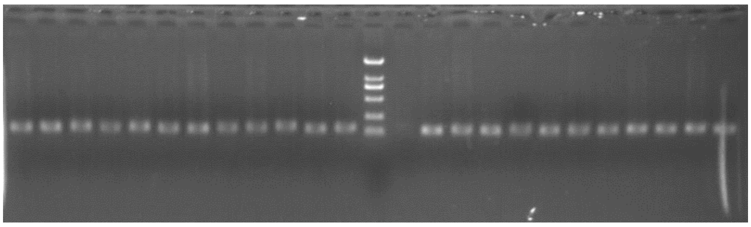

FORWARD PRIMER1 (5'-3')：ACAGTCGACCCAGCATTACC **REVERSE PRIMER1 (5'-3')**：AGCAACTGAAACCCACCATC

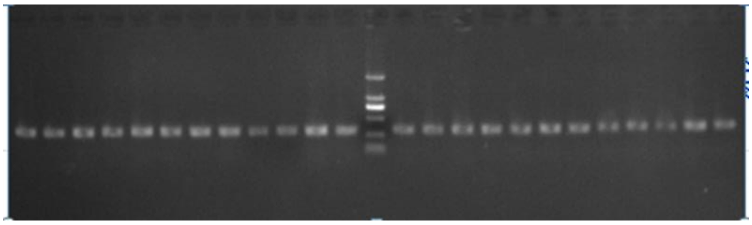

FORWARD PRIMER1 (5'-3')：TCTCAAATCACTTCCGGACC **REVERSE PRIMER1 (5'-3')**：ATCAGACGGCAACAGGAGAC

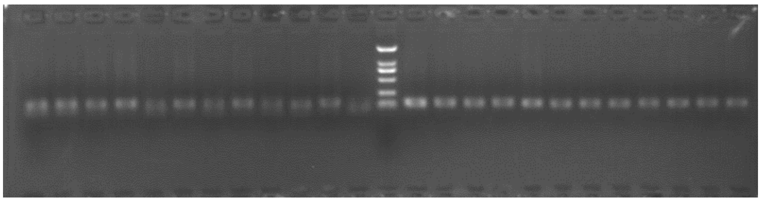

FORWARD PRIMER1 (5'-3')：AACAAACTGACCCCAAAACG **REVERSE PRIMER1 (5'-3')**：CCCCACCAAGAATTTTCAGA

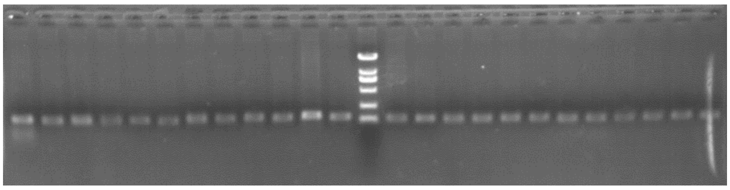

FORWARD PRIMER1 (5'-3')：TCCAGAGTCTTCCATTTGGG **REVERSE PRIMER1 (5'-3')**：CCCACCAAATAGCCAACAAC

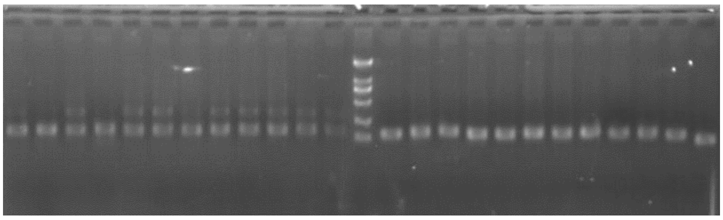

FORWARD PRIMER1 (5'-3')：GAAACCCCCTTCCTTACCAA **REVERSE PRIMER1 (5'-3')**：GAGGGCCATGAAGTTGAGAA

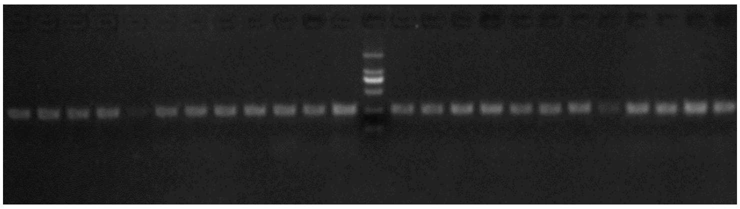

FORWARD PRIMER1 (5'-3')：TCGTAAAGCATGTCGTCGTC **REVERSE PRIMER1 (5'-3')**：GGAAGCACAGCAAGACATGA

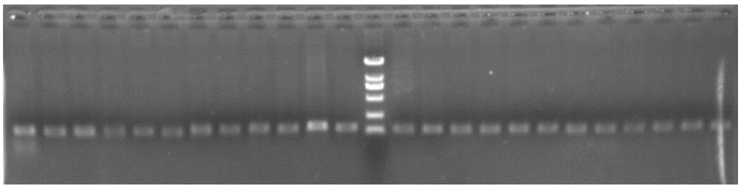

FORWARD PRIMER1 (5'-3'): GATCATGAGAGACCCGGAAA **REVERSE PRIMER1 (5'-3')**: AGCAATTTAACGGCGACATC

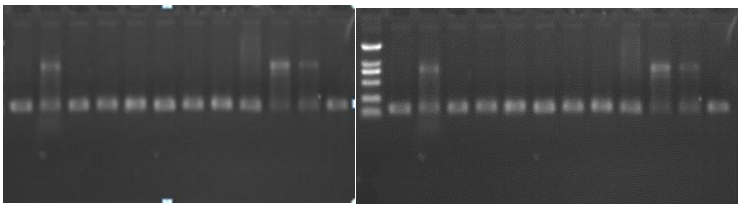

FORWARD PRIMER1 (5'-3'): CATGTCAGAGAGGGGTCTCC **REVERSE PRIMER1 (5'-3')**: CAGAGGTTCGGAAGAGCATC

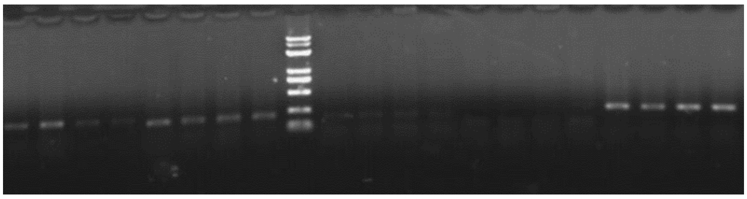

FORWARD PRIMER1 (5'-3'): GGCTTTGAAAATCCGATGAA **REVERSE PRIMER1 (5'-3')**: AGCCAGAGACACAAAAACCC

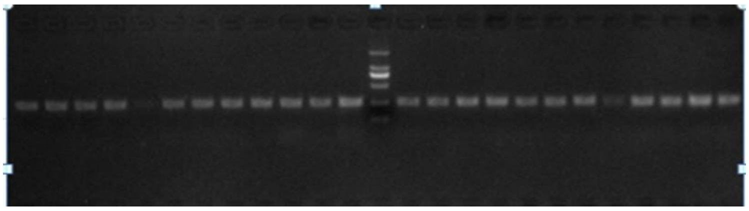

FORWARD PRIMER1 (5'-3'): CTGGCTTTTCAAGCACAGTCT **REVERSE PRIMER1 (5'-3')**: ATTTTTGCTTTGGGTTGTGG

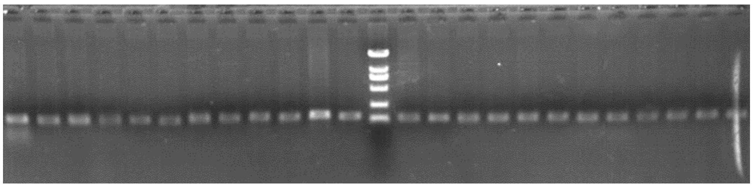

FORWARD PRIMER1 (5'-3'): CCAACGGTTCTAAACTCCCA **REVERSE PRIMER1 (5'-3')**: CAGAATTTGCAGAGCCTTCAG

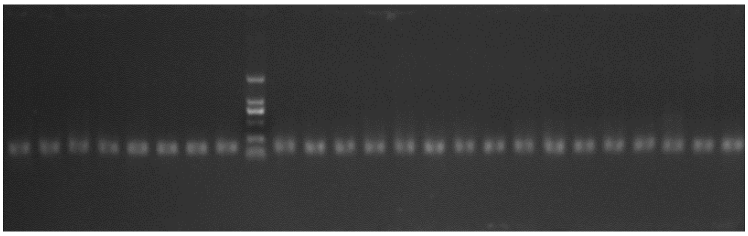

FORWARD PRIMER1 (5'-3'): TGGCTGACCAGTTCCTTCTT **REVERSE PRIMER1 (5'-3')**: AAATCCAACCCAACCCTACC

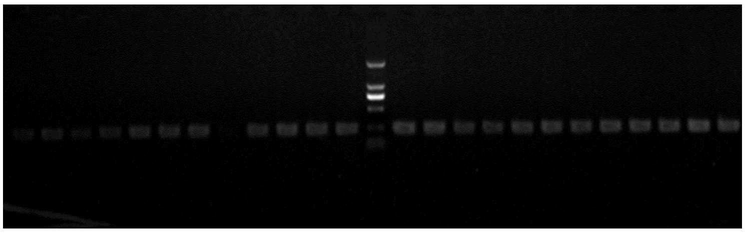

FORWARD PRIMER1 (5'-3'): TTGTGGAGCACTTAGCTCGAT **REVERSE PRIMER1 (5'-3')**: TGAAGACGATGATGATGGGA

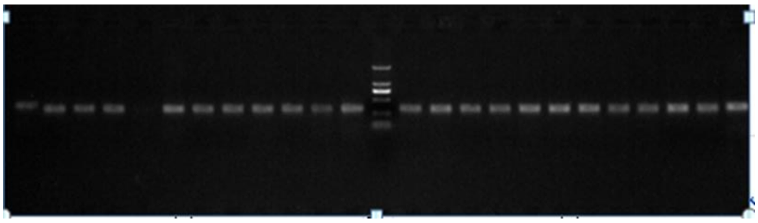

FORWARD PRIMER1 (5'-3'): CCTACGAGCGTCTCTTGACC **REVERSE PRIMER1 (5'-3')**: ATTGATAACCCACCCATCCA

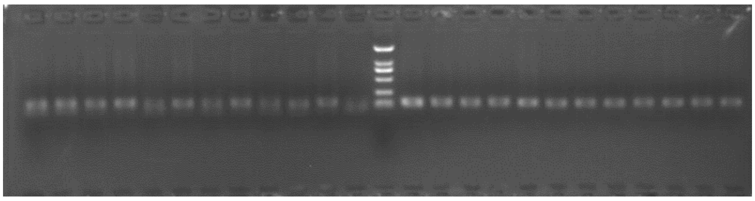

FORWARD PRIMER1 (5'-3'): CTGTCTCAGCTTCCAGCCTT **REVERSE PRIMER1 (5'-3')**: GGCTAAGACCACCACTTGGA

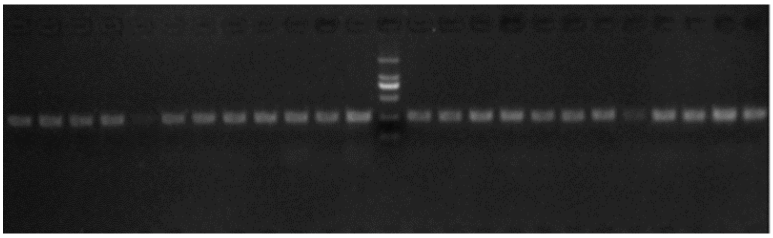

FORWARD PRIMER1 (5'-3'): AAGATCCACAGCCTTCAGGA **REVERSE PRIMER1 (5'-3')**: TCATCATCACCACCATCACC

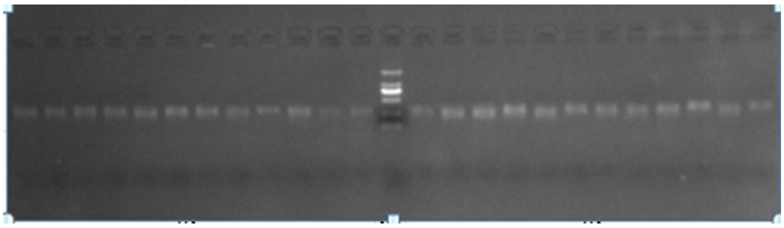

FORWARD PRIMER1 (5'-3'): GGGTTGTTCGAGGTCGTTTA **REVERSE PRIMER1 (5'-3')**: CAACGCTAACTTCACCAGCA

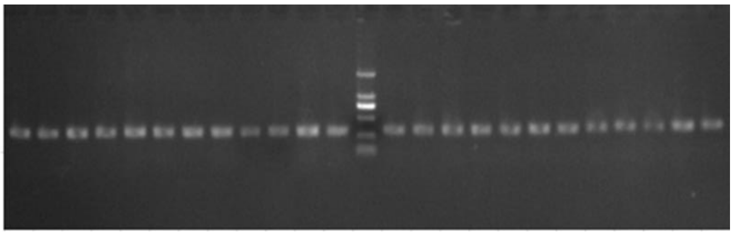

FORWARD PRIMER1 (5'-3'): ATTTTCATTTCCGTTCACGC **REVERSE PRIMER1 (5'-3'):** GGCCATGTCTAGTGGGTCTG

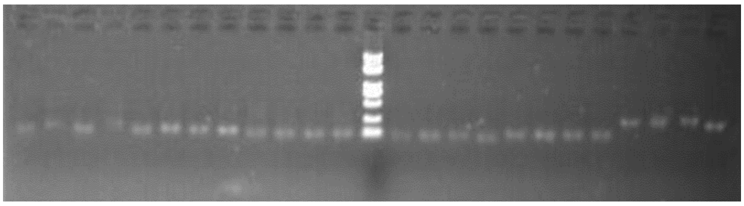

FORWARD PRIMER1 (5'-3'): GTGGCGGTCGTAATCTGTTT **REVERSE PRIMER1 (5'-3'):** CGTCTCCTCTTTCTGTTGCC

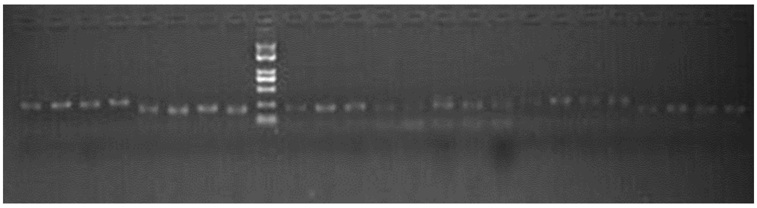

FORWARD PRIMER1 (5'-3'): ACCCACATGGAATCTGGAAG **REVERSE PRIMER1 (5'-3'):** TCCTTGTGACATGTGTTGGAA

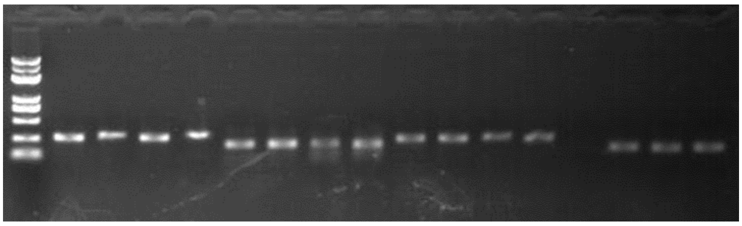

FORWARD PRIMER1 (5'-3'): CTGGCTTTTCAAGCACAGTCT **REVERSE PRIMER1 (5'-3'):** ATTTTTGCTTTGGGTTGTGG

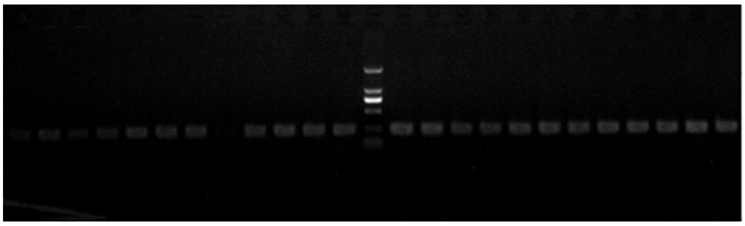

FORWARD PRIMER1 (5'-3'): GCCTCTATTGCCATGTTGGT **REVERSE PRIMER1 (5'-3'):** ATCATCCAGTGGCTGGAAAG

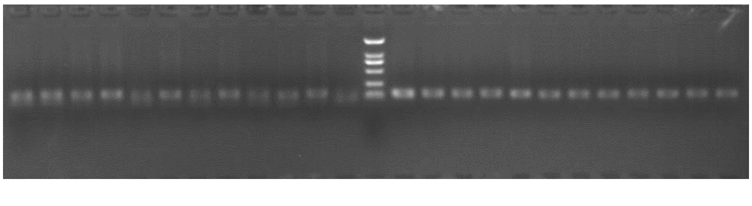

FORWARD PRIMER1 (5'-3'): TTAACTGGTGGTTGCCCTTC **REVERSE PRIMER1 (5'-3'):** GGCTCTTCTATTGGGATGGA

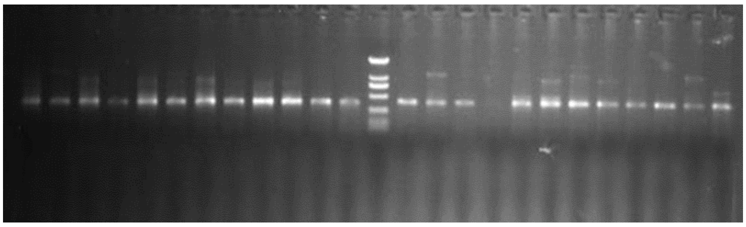

FORWARD PRIMER1 (5'-3')：CATCGTCCTCCAATTCGTTT **REVERSE PRIMER1 (5'-3')**：GCTTCTCCAATTTCCCCTTC

Supplementary Figure 3

UPGMA dendrogram based on the genetic distance showing the relationships between the 60 individuals

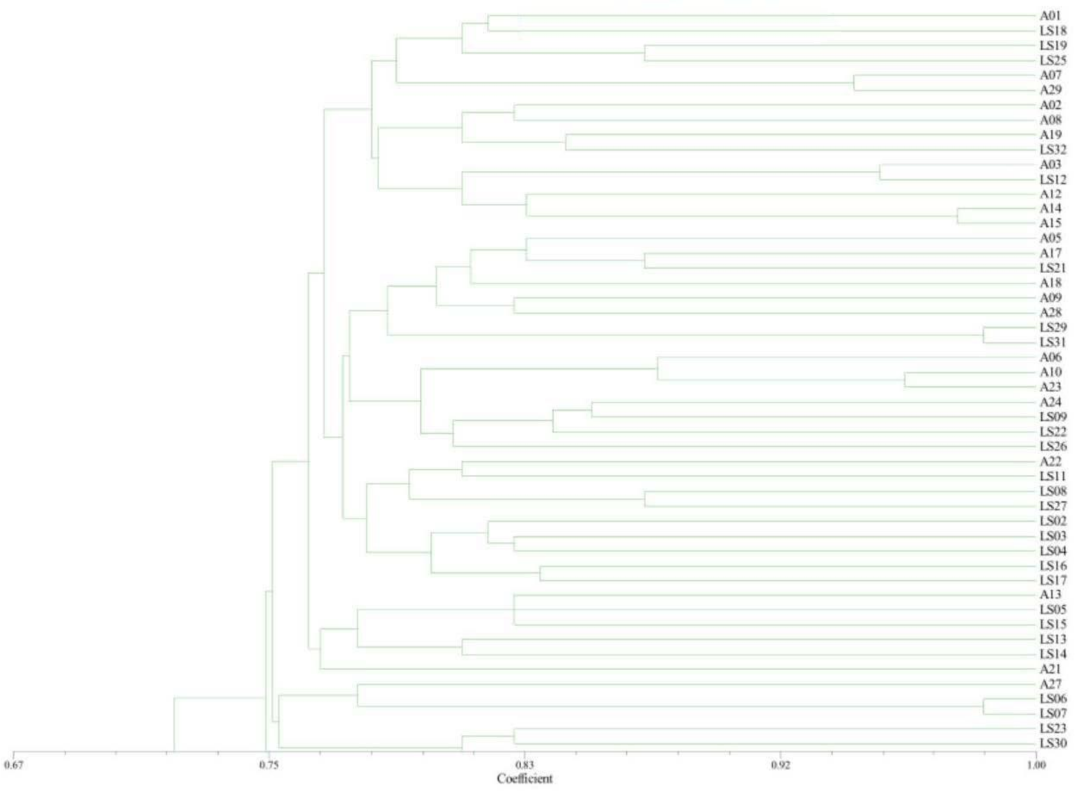

Supplementary Table 1

Species-based distribution of BLASTX matches for unigenes against NCBI Nr database

Species	Unigene number	Percent
Prunus mume	11565	43.471%
Malus domestica	3561	13.385%
Pyrus x bretschneideri	3189	11.987%
Cephalotus follicularis	829	3.116%
Fragaria vesca	793	2.981%
Theobroma cacao	603	2.267%
Anthurium amnicola	492	1.849%
Prunus persica	444	1.669%
Medicago truncatula	377	1.417%
Arabidopsis thaliana	298	1.120%
Gossypium arboreum	279	1.049%
Brassica napus	273	1.026%
Ziziphus jujuba	265	0.996%
Corchorus capsularis	237	0.891%
Morus notabilis	215	0.808%
Juglans regia	212	0.797%
Corchorus olitorius	189	0.710%
Populus trichocarpa	168	0.631%
Vitis vinifera	111	0.417%
Cajanus cajan	93	0.350%
Citrus sinensis	73	0.274%
Cynara cardunculus	71	0.267%
Ricinus communis	69	0.259%
Gossypium hirsutum	65	0.244%
Jatropha curcas	61	0.229%
Populus euphratica	59	0.222%
Noccaea caerulescens	58	0.218%
Glycine soja	55	0.207%
Glycine max	53	0.199%
Zea mays	53	0.199%
Oryza sativa Japonica Group	51	0.192%
Nelumbo nucifera	49	0.184%
Auxenochlorella protothecoides	47	0.177%
Eucalyptus grandis	46	0.173%
Lupinus angustifolius	41	0.154%
Dorcoceras hygrometricum	41	0.154%
Nicotiana attenuata	32	0.120%
Gossypium raimondii	31	0.117%

Cucumis melo	31	0.117%
Beta vulgaris	31	0.117%
Cucumis sativus	30	0.113%
Arachis duranensis	29	0.109%
Malus x robusta	28	0.105%
Arachis ipaensis	27	0.101%
Ipomoea nil	27	0.101%
Cicer arietinum	26	0.098%
Daucus carota	26	0.098%
Chrysochromulina sp. CCMP291	25	0.094%
Asparagus officinalis	25	0.094%
Klebsormidium flaccidum	23	0.086%
Prunus avium	23	0.086%
Coccomyxa subellipsoidea C-169	22	0.083%
Sesamum indicum	22	0.083%
Rosa rugosa	22	0.083%
Ananas comosus	20	0.075%
Arabidopsis lyrata	20	0.075%
Prunus pseudocerasus	20	0.075%
Solanum lycopersicum	19	0.071%
Phoenix dactylifera	18	0.068%
Nicotiana tabacum	18	0.068%
Eriobotrya japonica	18	0.068%
Vigna angularis	17	0.064%
Citrus limon	16	0.060%
Chlorella variabilis	16	0.060%
Malus prunifolia	16	0.060%
Nannochloropsis gaditana	15	0.056%
Vigna radiata	15	0.056%
Camelina sativa	14	0.053%
Tarenaya hassleriana	14	0.053%
Solanum tuberosum	14	0.053%
Amborella trichopoda	13	0.049%
Erythranthe guttata	13	0.049%
Elaeis guineensis	13	0.049%
Symbiodinium microadriaticum	12	0.045%
Ostreococcus tauri	12	0.045%
Galdieria sulphuraria	12	0.045%
Setaria italica	12	0.045%
Phaseolus vulgaris	11	0.041%
Musa acuminata	11	0.041%
Chondrus crispus	11	0.041%
Malus baccata	10	0.038%

Volvox carteri f. nagariensis	10	0.038%
Monoraphidium neglectum	10	0.038%
Prunus salicina	10	0.038%
Oryza brachyantha	10	0.038%
Lotus japonicus	10	0.038%
Nicotiana tomentosiformis	10	0.038%
Capsicum annuum	10	0.038%
Prunus armeniaca	9	0.034%
Prunus sibirica	9	0.034%
Thalassiosira pseudonana CCMP1335	9	0.034%
Chlamydomonas reinhardtii	9	0.034%
Pyrus communis	9	0.034%
Ectocarpus siliculosus	9	0.034%
Aegilops tauschii	8	0.030%
Fragilariopsis cylindrus CCMP1102	8	0.030%
Pyrus pyrifolia	8	0.030%
Zostera marina	8	0.030%
Cyanidioschyzon merolae	8	0.030%
Blastocystis sp. subtype 4	8	0.030%
Solanum pennellii	7	0.026%
Guillardia theta CCMP2712	7	0.026%
Prunus dulcis	7	0.026%
Emiliania huxleyi CCMP1516	7	0.026%
Triticum urartu	7	0.026%
Pyrus betulifolia	6	0.023%
Blastocystis sp. ATCC 50177/Nand II	6	0.023%
Nicotiana sylvestris	6	0.023%
Micromonas commoda	6	0.023%
Manihot esculenta	6	0.023%
Oryza sativa Indica Group	6	0.023%
Prunus cerasifera x Prunus munsoniana	6	0.023%
Vernicia fordii	5	0.019%
Malus hupehensis	5	0.019%
Raphanus sativus	5	0.019%
Hevea brasiliensis	5	0.019%
Bathycoccus prasinos	5	0.019%
Populus tomentosa	5	0.019%
Fragaria x ananassa	5	0.019%
Prunus serrulata	5	0.019%
Brassica oleracea	5	0.019%
Oryza minuta	4	0.015%
Dimocarpus longan	4	0.015%
Helicosporidium sp. ATCC 50920	4	0.015%

Prunus cerasoides	4	0.015%
Phaeodactylum tricornutum	4	0.015%
Brachypodium distachyon	4	0.015%
Brassica rapa	4	0.015%
Dichanthelium oligosanthes	4	0.015%
Pinus koraiensis	4	0.015%
Pisum sativum	4	0.015%
Carica papaya	4	0.015%
Arachis hypogaea	4	0.015%
Gracilariopsis lemaneiformis	4	0.015%
Helianthus annuus	4	0.015%
Genlisea aurea	4	0.015%
Pfiesteria piscicida	4	0.015%
Quercus suber	3	0.011%
Pentactina rupicola	3	0.011%
Adenostoma fasciculatum	3	0.011%
Chlorella pyrenoidosa	3	0.011%
Citrullus lanatus	3	0.011%
Panax ginseng	3	0.011%
Rosa hybrid cultivar	3	0.011%
Prunus cerasifera	3	0.011%
Chlorella sp. ArM0029B	3	0.011%
Sorbus aucuparia	3	0.011%
Ficus racemosa	3	0.011%
Vitis labrusca	3	0.011%
Populus alba	3	0.011%
Chlorella vulgaris	3	0.011%
Phaeodactylum tricornutum CCAP 1055/1	3	0.011%
Camellia sinensis	3	0.011%
Lingulodinium polyedrum	3	0.011%
Aureococcus anophagefferens	2	0.008%
Nannochloropsis gaditana CCMP526	2	0.008%
Malus hybrid cultivar	2	0.008%
Scopolia parviflora	2	0.008%
Antirrhinum majus	2	0.008%
Ulva linza	2	0.008%
Pyropia haitanensis	2	0.008%
Phyllostachys edulis	2	0.008%
Castanopsis concinna	2	0.008%
Polyblepharides amylifera	2	0.008%
Dunaliella salina	2	0.008%
Populus tremula	2	0.008%

Cenchrus americanus	2	0.008%
Cyanophora paradoxa	2	0.008%
Vernicia montana	2	0.008%
Malus zumi	2	0.008%
Rosa chinensis	2	0.008%
Hordeum vulgare	2	0.008%
Solanum demissum	2	0.008%
Camellia oleifera	2	0.008%
Acacia mangium	2	0.008%
Lagarosiphon major	2	0.008%
Dendrobium catenatum	2	0.008%
Morella rubra	2	0.008%
Prunus padus	2	0.008%
Triticum aestivum	2	0.008%
Selaginella moellendorffii	2	0.008%
Rhodomonas salina	2	0.008%
Prunus hypoleuca	2	0.008%
Prinsepia utilis	2	0.008%
Miscanthus sinensis	2	0.008%
Blastocystis hominis	2	0.008%
Pyrus calleryana	2	0.008%
Hypseocharis bilobata	2	0.008%
Prunus humilis	2	0.008%
Trichosanthes dioica	2	0.008%
Nicotiana benthamiana	2	0.008%
Geranium brycei	2	0.008%
Conocephalum conicum	2	0.008%
Rubus sp. 29c4-5	2	0.008%
Citrus x paradisi	2	0.008%
Roya obtusa	1	0.004%
Chlamydomonas incerta	1	0.004%
Sesuvium portulacastrum	1	0.004%
Bryonia dioica	1	0.004%
Coffea tetragona	1	0.004%
Endiandra globosa	1	0.004%
Guillardia theta	1	0.004%
Synura petersenii	1	0.004%
Prunus sogdiana	1	0.004%
Monomastix sp. OKE-1	1	0.004%
Pyramimonas cordata	1	0.004%
Cynanchum auriculatum	1	0.004%
Gossypium barbadense	1	0.004%
Cyanidium caldarium	1	0.004%

Mentha spicata	1	0.004%
Prunus tenella	1	0.004%
Pinus palustris	1	0.004%
Tetraselmis chuii	1	0.004%
Gonium pectorale	1	0.004%
Cecropia obtusifolia	1	0.004%
Striga asiatica	1	0.004%
Phalaenopsis aphrodite	1	0.004%
Oryza latifolia	1	0.004%
Corylus avellana	1	0.004%
Prunus cerasus	1	0.004%
Taraxacum kok-saghyz	1	0.004%
Mesembryanthemum crystallinum	1	0.004%
Prunus yedoensis	1	0.004%
Auxenochlorella pyrenoidosa	1	0.004%
Papaver somniferum	1	0.004%
Fragaria nipponica	1	0.004%
Gossypium herbaceum	1	0.004%
Cynomorium coccineum	1	0.004%
Sorghum bicolor	1	0.004%
Masdevallia picturata	1	0.004%
Populus laurifolia	1	0.004%
Erigeron bellidiastrum	1	0.004%
Ostreococcus lucimarinus CCE9901	1	0.004%
Ulota hutchinsiae	1	0.004%
Barringtonia racemosa	1	0.004%
Medicago sativa	1	0.004%
Citrus unshiu	1	0.004%
Glycine tomentella	1	0.004%
Digitalis lanata	1	0.004%
Prunus domestica	1	0.004%
Treubia lacunosa	1	0.004%
Chlamydomonas moewusii	1	0.004%
Vitis pseudoreticulata	1	0.004%
Prunus cerasus x Prunus canescens	1	0.004%
Pinus balfouriana	1	0.004%
Quercus aliena	1	0.004%
Saintpaulia hybrid cultivar	1	0.004%
Cydonia oblonga	1	0.004%
Eustoma exaltatum	1	0.004%
Lagerstroemia floribunda	1	0.004%
Chaenomeles sinensis	1	0.004%
Chrysanthemum x morifolium	1	0.004%

Ampelopsis glandulosa	1	0.004%
Dahlia pinnata	1	0.004%
Neillia thibetica	1	0.004%
Marchantia polymorpha	1	0.004%
Hyacinthus orientalis	1	0.004%
Isochrysis galbana	1	0.004%
Goniomonas truncata	1	0.004%
Symphyocladia latiuscula	1	0.004%
Salvia miltiorrhiza	1	0.004%
Macrocystis pyrifera	1	0.004%
Populus x canadensis	1	0.004%
Ephedra equisetifolia	1	0.004%
Craterostigma plantagineum	1	0.004%
Eleusine coracana	1	0.004%
Physalis peruviana	1	0.004%
Fragaria moschata	1	0.004%
Malus orientalis	1	0.004%
Coffea canephora	1	0.004%
Choreocolax polysiphoniae	1	0.004%
Sambucus nigra	1	0.004%
Aeschynomene denticulata	1	0.004%
Cypripedium formosanum	1	0.004%
Pteridium aquilinum	1	0.004%
Ulva fasciata	1	0.004%
Potentilla tucumanensis	1	0.004%
Aulacantha scolymantha	1	0.004%
Picea glauca	1	0.004%
Rhododendron formosanum	1	0.004%
Pyrus ussuriensis	1	0.004%
Citrus clementina	1	0.004%
Linum usitatissimum	1	0.004%
Asclepias syriaca	1	0.004%
Sargassum muticum	1	0.004%
Oryza sativa	1	0.004%
Spinacia oleracea	1	0.004%
Pyrus x bretschneideri x Pyrus pyrifolia	1	0.004%
Erodium crassifolium	1	0.004%
Vicia sativa	1	0.004%
Silene paradoxa	1	0.004%
Musa AB Group	1	0.004%
Melianthus villosus	1	0.004%
Rhododendron kanehirae	1	0.004%
Rhus chinensis	1	0.004%

Fraxinus excelsior	1	0.004%
Vitis quinquangularis	1	0.004%
Nymphaea hybrid cultivar	1	0.004%
Rosa roxburghii	1	0.004%
Tarenaya spinosa	1	0.004%
Oryza longistaminata	1	0.004%
Arabis alpina	1	0.004%
Ipomoea batatas	1	0.004%
Melilotus albus	1	0.004%
Physcomitrella patens	1	0.004%
Hildenbrandia rubra	1	0.004%
Pyracantha coccinea	1	0.004%
Puccinellia tenuiflora	1	0.004%
Olea europaea	1	0.004%
Nicotiana tabacum/Hyoscyamus niger cybrid	1	0.004%
Elaeagnus umbellata	1	0.004%
Potamophila parviflora	1	0.004%
Populus davidiana x Populus alba var. pyramidalis	1	0.004%
Lactuca sativa	1	0.004%
Prunus subhirtella	1	0.004%
Viburnum amplificatum	1	0.004%
Eurya emarginata	1	0.004%
Thalassiosira oceanica	1	0.004%
Malpighia glabra	1	0.004%
Pandanus utilis	1	0.004%
Lycoris aurea	1	0.004%
Spathelia splendens	1	0.004%
Vicia faba	1	0.004%
Malus sieversii	1	0.004%
Solanum melongena	1	0.004%
Oenothera elata	1	0.004%
Vaccinium macrocarpon	1	0.004%
Stellaria longipes	1	0.004%
Dunaliella bioculata	1	0.004%
Quercus robur	1	0.004%
Pterocladia media	1	0.004%
Morus alba	1	0.004%
Rhodomonas sp. CCMP768	1	0.004%
Liriodendron tulipifera	1	0.004%
Cannabis sativa	1	0.004%
Chlorochytrium lemnae	1	0.004%

Pistacia vera	1	0.004%
Rubus occidentalis	1	0.004%
Brachiaria hybrid cultivar	1	0.004%
Thraustochytrium aureum	1	0.004%
Diospyros kaki	1	0.004%
Chlamydomonas sp. ICE-L	1	0.004%
Pottingeria acuminata	1	0.004%

Supplementary Table 2

Summary of GO term assignment for *S. incisa* transcriptome

Go type	Term	Unigene counts	Percent (%)
Biological Process	reproduction	369	0.705%
	cell killing	1	0.002%
	immune system process	84	0.160%
	metabolic process	6195	11.832%
	cellular process	5517	10.537%
	reproductive process	366	0.699%
	biological adhesion	29	0.055%
	signaling	428	0.817%
	multicellular organismal process	583	1.114%
	developmental process	762	1.455%
	growth	101	0.193%
	locomotion	3	0.006%
	single-organism process	4179	7.982%
	rhythmic process	15	0.029%
	positive regulation of biological process	126	0.241%
	negative regulation of biological process	186	0.355%
	regulation of biological process	1529	2.920%
	response to stimulus	1334	2.548%
	localization	1372	2.621%
	multi-organism process	189	0.361%
	biological regulation	1676	3.201%
	cellular component organization or biogenesis	1020	1.948%
	detoxification	7	0.013%
Molecular Function	transcription factor activity, protein binding	8	0.015%
	nucleic acid binding transcription factor activity	140	0.267%
	catalytic activity	5706	10.898%
	signal transducer activity	67	0.128%
	structural molecule activity	160	0.306%
	transporter activity	494	0.944%
	binding	4362	8.331%
	electron carrier activity	7	0.013%
	antioxidant activity	28	0.053%
	molecular transducer activity	43	0.082%
	molecular function regulator	41	0.078%
Cellular Component	extracellular region	75	0.143%
	cell	3401	6.496%
	nucleoid	1	0.002%
	membrane	1918	3.663%

	virion	35	0.067%
	cell junction	139	0.265%
	extracellular matrix	2	0.004%
	membrane-enclosed lumen	66	0.126%
	macromolecular complex	1049	2.004%
	organelle	2531	4.834%
	extracellular region part	3	0.006%
	organelle part	1093	2.088%
	virion part	35	0.067%
	membrane part	1476	2.819%
	cell part	3401	6.496%
	supramolecular fiber	4	0.008%

Supplementary Table3

A: KOG annotation of *S.incisa* unigenes

**KOG categories**	**KOG description**	**Number**	**Percent (%)**
A	RNA processing and modification	1221	5.429%
B	Chromatin structure and dynamics	356	1.583%
C	Energy production and conversion	992	4.411%
D	Cell cycle control, cell division, chromosome partitioning	573	2.548%
E	Amino acid transport and metabolism	671	2.984%
F	Nucleotide transport and metabolism	217	0.965%
G	Carbohydrate transport and metabolism	885	3.935%
H	Coenzyme transport and metabolism	179	0.796%
I	Lipid transport and metabolism	820	3.646%
J	Translation, ribosomal structure and biogenesis	1274	5.665%
K	Transcription	1227	5.456%
L	Replication, recombination and repair	749	3.331%
M	Cell wall/membrane/envelope biogenesis	232	1.032%
N	Cell motility	17	0.076%
O	Posttranslational modification, protein turnover, chaperones	2540	11.294%
P	Inorganic ion transport and metabolism	543	2.415%
Q	Secondary metabolites biosynthesis, transport and catabolism	691	3.073%
R	General function prediction only	3775	16.786%
S	Function unknown	948	4.215%
T	Signal transduction mechanisms	2593	11.530%
U	Intracellular trafficking, secretion, and vesicular transport	972	4.322%
V	Defense mechanisms	154	0.685%
W	Extracellular structures	80	0.356%
Y	Nuclear structure	62	0.276%
Z	Cytoskeleton	718	3.193%

B: Summary of KEGG pathways involved in the *S.incisa* transcriptome

Number	Pathway ID	Pathway	Unigene number	Percent (%)
1	ko01100	Metabolic pathways	2193	17.644 %
2	ko01110	Biosynthesis of secondary metabolites	1137	9.148%
3	ko03010	Ribosome	540	4.345%
4	ko04626	Plant-pathogen interaction	382	3.073%
5	ko01200	Carbon metabolism	297	2.390%
6	ko04141	Protein processing in endoplasmic reticulum	262	2.108%
7	ko01230	Biosynthesis of amino acids	257	2.068%
8	ko03040	Spliceosome	251	2.019%
9	ko00230	Purine metabolism	227	1.826%
10	ko03013	RNA transport	227	1.826%
11	ko04075	Plant hormone signal transduction	227	1.826%
12	ko00190	Oxidative phosphorylation	195	1.569%
13	ko04144	Endocytosis	185	1.488%
14	ko00240	Pyrimidine metabolism	168	1.352%
15	ko04016	MAPK signaling pathway - plant	160	1.287%
16	ko04120	Ubiquitin mediated proteolysis	160	1.287%
17	ko00010	Glycolysis / Gluconeogenesis	154	1.239%
18	ko03018	RNA degradation	138	1.110%
19	ko03015	mRNA surveillance pathway	137	1.102%
20	ko00500	Starch and sucrose metabolism	135	1.086%
21	ko04145	Phagosome	123	0.990%
22	ko03008	Ribosome biogenesis in eukaryotes	120	0.965%
23	ko03440	Homologous recombination	118	0.949%
24	ko00270	Cysteine and methionine metabolism	115	0.925%
25	ko00940	Phenylpropanoid biosynthesis	115	0.925%
26	ko00564	Glycerophospholipid metabolism	110	0.885%
27	ko00620	Pyruvate metabolism	106	0.853%
28	ko00520	Amino sugar and nucleotide sugar metabolism	105	0.845%
29	ko00480	Glutathione metabolism	97	0.780%
30	ko04146	Peroxisome	97	0.780%
31	ko03420	Nucleotide excision repair	96	0.772%
32	ko00710	Carbon fixation in photosynthetic organisms	94	0.756%
33	ko03030	DNA replication	93	0.748%
34	ko00630	Glyoxylate and dicarboxylate metabolism	89	0.716%
35	ko00561	Glycerolipid metabolism	86	0.692%

36	ko01212	Fatty acid metabolism	80	0.644%
37	ko03430	Mismatch repair	80	0.644%
38	ko00195	Photosynthesis	73	0.587%
39	ko00970	Aminoacyl-tRNA biosynthesis	73	0.587%
40	ko00260	Glycine, serine and threonine metabolism	72	0.579%
41	ko02010	ABC transporters	70	0.563%
42	ko03410	Base excision repair	69	0.555%
43	ko00020	Citrate cycle (TCA cycle)	67	0.539%
44	ko04070	Phosphatidylinositol signaling system	67	0.539%
45	ko00051	Fructose and mannose metabolism	66	0.531%
46	ko00562	Inositol phosphate metabolism	66	0.531%
47	ko00040	Pentose and glucuronate interconversions	65	0.523%
48	ko00330	Arginine and proline metabolism	65	0.523%
49	ko00250	Alanine, aspartate and glutamate metabolism	64	0.515%
50	ko03050	Proteasome	61	0.491%
51	ko03020	RNA polymerase	60	0.483%
52	ko04136	Autophagy - other eukaryotes	60	0.483%
53	ko03022	Basal transcription factors	59	0.475%
54	ko00030	Pentose phosphate pathway	57	0.459%
55	ko01210	2-Oxocarboxylic acid metabolism	56	0.451%
56	ko03060	Protein export	55	0.443%
57	ko00053	Ascorbate and aldarate metabolism	54	0.434%
58	ko00280	Valine, leucine and isoleucine degradation	54	0.434%
59	ko00400	Phenylalanine, tyrosine and tryptophan biosynthesis	54	0.434%
60	ko00510	N-Glycan biosynthesis	54	0.434%
61	ko00071	Fatty acid degradation	52	0.418%
62	ko00860	Porphyrin and chlorophyll metabolism	51	0.410%
63	ko00900	Terpenoid backbone biosynthesis	50	0.402%
64	ko04712	Circadian rhythm - plant	50	0.402%
65	ko00052	Galactose metabolism	47	0.378%
66	ko00310	Lysine degradation	47	0.378%
67	ko00941	Flavonoid biosynthesis	46	0.370%
68	ko00061	Fatty acid biosynthesis	45	0.362%
69	ko00062	Fatty acid elongation	45	0.362%
70	ko00410	beta-Alanine metabolism	45	0.362%
71	ko00130	Ubiquinone and other terpenoid-quinone biosynthesis	44	0.354%
72	ko00460	Cyanoamino acid metabolism	43	0.346%
73	ko04130	SNARE interactions in vesicular transport	43	0.346%
74	ko00592	alpha-Linolenic acid metabolism	42	0.338%
75	ko00350	Tyrosine metabolism	41	0.330%

76	ko00640	Propanoate metabolism	40	0.322%
77	ko00220	Arginine biosynthesis	38	0.306%
78	ko00905	Brassinosteroid biosynthesis	37	0.298%
79	ko00565	Ether lipid metabolism	36	0.290%
80	ko00360	Phenylalanine metabolism	34	0.274%
81	ko00563	Glycosylphosphatidylinositol(GPI)-anchor biosynthesis	34	0.274%
82	ko00920	Sulfur metabolism	34	0.274%
83	ko00906	Carotenoid biosynthesis	33	0.266%
84	ko00380	Tryptophan metabolism	32	0.257%
85	ko00650	Butanoate metabolism	32	0.257%
86	ko00910	Nitrogen metabolism	32	0.257%
87	ko00600	Sphingolipid metabolism	31	0.249%
88	ko00100	Steroid biosynthesis	30	0.241%
89	ko00770	Pantothenate and CoA biosynthesis	30	0.241%
90	ko01040	Biosynthesis of unsaturated fatty acids	30	0.241%
91	ko00760	Nicotinate and nicotinamide metabolism	29	0.233%
92	ko00790	Folate biosynthesis	28	0.225%
93	ko00196	Photosynthesis - antenna proteins	27	0.217%
94	ko00945	Stilbenoid, diarylheptanoid and gingerol biosynthesis	26	0.209%
95	ko00511	Other glycan degradation	25	0.201%
96	ko00670	One carbon pool by folate	25	0.201%
97	ko00730	Thiamine metabolism	24	0.193%
98	ko00950	Isoquinoline alkaloid biosynthesis	24	0.193%
99	ko00073	Cutin, suberine and wax biosynthesis	22	0.177%
100	ko00590	Arachidonic acid metabolism	22	0.177%
101	ko00909	Sesquiterpenoid and triterpenoid biosynthesis	22	0.177%
102	ko00531	Glycosaminoglycan degradation	21	0.169%
103	ko00960	Tropane, piperidine and pyridine alkaloid biosynthesis	21	0.169%
104	ko00740	Riboflavin metabolism	20	0.161%
105	ko00290	Valine, leucine and isoleucine biosynthesis	18	0.145%
106	ko00340	Histidine metabolism	18	0.145%
107	ko00591	Linoleic acid metabolism	18	0.145%
108	ko00750	Vitamin B6 metabolism	18	0.145%
109	ko00780	Biotin metabolism	18	0.145%
110	ko00908	Zeatin biosynthesis	18	0.145%
111	ko00450	Selenocompound metabolism	17	0.137%
112	ko00904	Diterpenoid biosynthesis	17	0.137%
113	ko03450	Non-homologous end-joining	16	0.129%
114	ko00300	Lysine biosynthesis	13	0.105%

115	ko00514	Other types of O-glycan biosynthesis	13	0.105%
116	ko00430	Taurine and hypotaurine metabolism	12	0.097%
117	ko00902	Monoterpenoid biosynthesis	12	0.097%
118	ko04122	Sulfur relay system	12	0.097%
119	ko00261	Monobactam biosynthesis	9	0.072%
120	ko00966	Glucosinolate biosynthesis	9	0.072%
121	ko00440	Phosphonate and phosphinate metabolism	8	0.064%
122	ko00603	Glycosphingolipid biosynthesis - globo and isoglobo series	8	0.064%
123	ko00901	Indole alkaloid biosynthesis	7	0.056%
124	ko00660	C5-Branched dibasic acid metabolism	6	0.048%
125	ko00785	Lipoic acid metabolism	6	0.048%
126	ko00943	Isoflavonoid biosynthesis	6	0.048%
127	ko00072	Synthesis and degradation of ketone bodies	5	0.040%
128	ko00232	Caffeine metabolism	4	0.032%
129	ko00604	Glycosphingolipid biosynthesis - ganglio series	3	0.024%
130	ko00942	Anthocyanin biosynthesis	3	0.024%
131	ko00944	Flavone and flavonol biosynthesis	3	0.024%
132	ko00965	Betalain biosynthesis	3	0.024%
133	ko00601	Glycosphingolipid biosynthesis - lacto and neolacto series	2	0.016%

Supplementary Table 4

Length distribution of the EST-SSRs of *S. incisa* based on the number of nucleotides repeat units

**Repeats**	**Di-**	**Tri-**	**Tetra-**	**Penta-**	**Hexa-**	**Total**	**Percentage (%)**
4	0	0	160	93	218	475	8.55
5	0	996	54	13	74	1137	20.47
6	799	467	28	10	13	1317	23.71
7	497	259	10	0	8	774	13.93
8	381	69	3	1	0	454	8.17
9	380	34	1	0	0	415	7.47
10	304	59	2	0	0	365	6.57
11	194	7	0	0	0	201	3.62
12	30	15	0	0	0	45	0.81
13	3	6	0	1	0	10	0.18
14	7	2	0	0	0	9	0.16
≥15	347	10	0	0	0	357	6.43
Total	2942	1924	258	118	313	5555	
Percentage (%)	52.96	34.64	4.64	2.12	5.63		

Supplementary Table 5

Detailed information of EST-SSRs based on the number of nt repeat unit in *S. incisa*

Repeats	4	5	6	7	8	9	10	11	12	13	14	15	>=16	total	Percentage (%)
AC/GT	-	-	58	27	18	7	12	9	4	2	1	3	6	147	2.646%
AG/CT	-	-	704	454	362	371	292	185	26	1	6	48	290	2739	49.307%
AT/AT	-	-	34	14	1	2								51	0.918%
CG/CG	-	-	3	2										5	0.090%
AAC/GTT	-	83	37	29	7	3	6	1	2		1			169	3.042%
AAG/CTT	-	252	129	86	7	11	22	4	9	3	1	2	5	531	9.559%
AAT/ATT	-	11	3	1										15	0.270%
ACC/GGT	-	146	70	36	7	1	6			3		1		270	4.860%
ACG/CGT	-	46	18	7	4	3	1							79	1.422%
ACT/AGT	-	22	7	2	1								1	33	0.594%
AGC/CTG	-	160	90	30	9	3	6		1				1	300	5.401%
AGG/CCT	-	104	51	31	17	3	6	1						213	3.834%
ATC/ATG	-	111	47	30	12	9	11	1	3					224	4.032%
CCG/CGG	-	61	15	7	5	1	1							90	1.620%
AAAC/GTTT	10	5	2	1										18	0.324%
AAAG/CTTT	37	12	6	1										56	1.008%
AAAT/ATTT	3													3	0.054%
AACC/GGTT	7	3												10	0.180%
AACG/CGTT		1												1	0.018%
AACT/AGTT	3	1												4	0.072%
AAGC/CTTG	6	1												7	0.126%
AAGG/CCTT	6	1	2											9	0.162%
AAGT/ACTT	1													1	0.018%
AATC/ATTG	5	1	4	1										11	0.198%
AATG/ATTC	4	2	1											7	0.126%
AATT/AATT	7	1												8	0.144%
ACAG/CTGT	7	5	2				1							15	0.270%
ACAT/ATGT	4	1	3	1			1							10	0.180%
ACCC/GGGT	1													1	0.018%
ACCG/CGGT	2	1												3	0.054%
ACCT/AGGT	1													1	0.018%
ACGC/CGTG	2	4		1	1									8	0.144%
ACGG/CCGT	1													1	0.018%
ACGT/ACGT	1													1	0.018%
ACTC/AGTG	6	2	2	2	1									13	0.234%
ACTG/AGTC	1	4				1								6	0.108%
AGAT/ATCT	5		1											6	0.108%
AGCC/CTGG	4	1		1										6	0.108%

AGCG/CGCT	5	3										8	0.144%
AGCT/AGCT	17	2										19	0.342%
AGGC/CCTG	3	1	2									6	0.108%
AGGG/CCCT	5	1	2		1							9	0.162%
ATCC/ATGG		1	1	2								4	0.072%
ATCG/ATCG	4											4	0.072%
ATGC/ATGC	2											2	0.036%
AAAAC/GTTTT	3	2	1									6	0.108%
AAAAG/CTTTT	9	2	1									12	0.216%
AAACC/GGTTT	1											1	0.018%
AAAGC/CTTTG	4											4	0.072%
AAAGG/CCTTT	1	1										2	0.036%
AAATC/ATTTG	2											2	0.036%
AAATG/ATTTC	2											2	0.036%
AACAC/GTGTT	2											2	0.036%
AACAG/CTGTT		2										2	0.036%
AACTC/AGTTG	1											1	0.018%
AAGAG/CTCTT	4	1	2									7	0.126%
AAGCC/CTTGG	4		1		1							6	0.108%
AAGCT/AGCTT	2											2	0.036%
AAGGC/CCTTG	1											1	0.018%
AAGGG/CCCTT	4		2									6	0.108%
AAGTC/ACTTG	1											1	0.018%
AATAG/ATTCT			1									1	0.018%
AATCC/ATTGG	8											8	0.144%
AATGG/ATTCC	4											4	0.072%
AATTC/AATTG	1											1	0.018%
ACACC/GGTGT	3											3	0.054%
ACAGT/ACTGT	2											2	0.036%
ACATC/ATGTG	1											1	0.018%
ACCAG/CTGGT	1											1	0.018%
ACCCC/GGGGT	1											1	0.018%
ACCGC/CGGTG	1											1	0.018%
ACCTC/AGGTG			1									1	0.018%
ACCTG/AGGTC	1	1										2	0.036%
ACGAG/CGTCT	4	1	1									6	0.108%
ACGCC/CGTGG	1											1	0.018%
ACTGC/AGTGC	2											2	0.036%
AGAGC/CTCTG	4											4	0.072%
AGAGG/CCTCT	5											5	0.090%
AGATC/ATCTG		1										1	0.018%
AGATG/ATCTC	3											3	0.054%
AGCCG/CGGCT	1	1										2	0.036%
AGCTC/AGCTG	4									1		5	0.090%

AGGCG/CCTCG	3			3	0.054%
AGGGC/CCCTG	1			1	0.018%
ATGCC/ATGGC	1	1		2	0.036%
AAAAAC/GTTTTT	2	3		5	0.090%
AAAAAG/CTTTTT	4			4	0.072%
AAAACC/GGTTTT	4			4	0.072%
AAAACG/CGTTTT	1	1		2	0.036%
AAAAGG/CCTTTT	2			2	0.036%
AAAAGT/ACTTTT	1			1	0.018%
AAAATC/ATTTTG	2	1		3	0.054%
AAAATG/ATTTTC	1			1	0.018%
AAACAC/GTGTTT	2			2	0.036%
AAACAG/CTGTTT		1	1	2	0.036%
AAACCC/GGGTTT	3			3	0.054%
AAACCG/CGGTTT	1			1	0.018%
AAACTC/AGTTTG	2	1		3	0.054%
AAACTG/AGTTTC	1			1	0.018%
AAAGAG/CTCTTT	2			2	0.036%
AAAGCC/CTTTGG	2			2	0.036%
AAAGGC/CCTTTG	1			1	0.018%
AAATAC/ATTTGT	1			1	0.018%
AAATCC/ATTTGG	6			6	0.108%
AAATCG/ATTTCG	1			1	0.018%
AAATGC/ATTTGC	1			1	0.018%
AAATGG/ATTTCC	1	1		2	0.036%
AAATTC/AATTTG	1			1	0.018%
AAATTG/AATTTC	1			1	0.018%
AACACC/GGTGTT	1	1		2	0.036%
AACAGC/CTGTTG	2	4	1	7	0.126%
AACATC/ATGTTG		1		1	0.018%
AACATG/ATGTTC	1			1	0.018%
AACCAC/GGTTGT	2			2	0.036%
AACCAG/CTGGTT	1	1		2	0.036%
AACCAT/ATGGTT	1			1	0.018%
AACCCC/GGGGTT		1		1	0.018%
AACCCG/CGGGTT		1		1	0.018%
AACCCT/AGGGTT	2			2	0.036%
AACCGC/CGGTTG		1		1	0.018%
AACCGG/CCGGTT	2			2	0.036%
AACCTC/AGGTTG		2		2	0.036%
AACCTG/AGGTTC	1			1	0.018%
AACGCC/CGTTGG	2			2	0.036%
AACGGC/CCGTTG			1	1	0.018%
AACTAC/AGTTGT		1		1	0.018%

AACTAG/AGTTCT	1				1	0.018%
AACTCC/AGTTGG	2	1			3	0.054%
AACTCG/AGTTCG	1				1	0.018%
AACTGC/AGTTGC	1				1	0.018%
AACTGG/AGTTCC	1				1	0.018%
AACTTC/AAGTTG	3				3	0.054%
AACTTG/AAGTTC	2		1		3	0.054%
AAGAGC/CTCTTG	1				1	0.018%
AAGAGG/CCTCTT	2	1			3	0.054%
AAGATG/ATCTTC	5	3			8	0.144%
AAGCAC/CTTGTG	1				1	0.018%
AAGCAG/CTGCTT	6	1			7	0.126%
AAGCAT/ATGCTT	1				1	0.018%
AAGCCC/CTTGGG	3				3	0.054%
AAGCCG/CGGCTT	1				1	0.018%
AAGCTC/AGCTTG		1			1	0.018%
AAGGAC/CCTTGT	3				3	0.054%
AAGGAG/CCTTCT	1				1	0.018%
AAGGGC/CCCTTG	1				1	0.018%
AAGGGG/CCCCTT		2			2	0.036%
AAGGTG/ACCTTC	1				1	0.018%
AAGTAG/ACTTCT	3				3	0.054%
AAGTGG/ACTTCC	1	1			2	0.036%
AATCAG/ATTCTG	1				1	0.018%
AATCCC/ATTGGG	2	1			3	0.054%
AATCGG/ATTCCG	1	1			2	0.036%
AATCTC/AGATTG	1				1	0.018%
AATCTG/AGATTC	1				1	0.018%
AATGGC/ATTGCC	3				3	0.054%
AATGGG/ATTCCC	8	1			9	0.162%
AATGGT/ACCATT	3				3	0.054%
AATGTG/ACATTC	1	1			2	0.036%
AATTAC/AATTGT	1				1	0.018%
AATTCC/AATTGG	2	1			3	0.054%
ACACAG/CTGTGT	1				1	0.018%
ACACCC/GGGTGT	2			1	3	0.054%
ACACCG/CGGTGT	1			1	2	0.036%
ACAGAG/CTCTGT	3	2		1	6	0.108%
ACAGCC/CTGTGG	1	1	1		3	0.054%
ACAGGG/CCCTGT	3				3	0.054%
ACAGTC/ACTGTG		1			1	0.018%
ACATGC/ATGTGC	1				1	0.018%
ACATGG/ATGTCC	1				1	0.018%
ACCACG/CGTGGT	3	1	1		5	0.090%

ACCACT/AGTGGT	1				1	0.018%
ACCAGC/CTGGTG	2		1		3	0.054%
ACCAGG/CCTGGT		1			1	0.018%
ACCATC/ATGGTG	5	1			6	0.108%
ACCATG/ATGGTC		2	1		3	0.054%
ACCCCG/CGGGGT	1				1	0.018%
ACCCTC/AGGGTG	2	2	1		5	0.090%
ACCGAG/CGGTCT	1				1	0.018%
ACCGCC/CGGTGG	6	2	1		9	0.162%
ACCTCC/AGGTGG	4				4	0.072%
ACCTCG/AGGTCG	1				1	0.018%
ACCTCT/AGAGGT	2				2	0.036%
ACCTGC/AGGTGC	3				3	0.054%
ACGATC/ATCGTG	1				1	0.018%
ACGATG/ATCGTC	1	1			2	0.036%
ACGCAG/CGTCTG	1				1	0.018%
ACGCCC/CGTGGG	2	1			3	0.054%
ACGGAG/CCGTCT		1			1	0.018%
ACGGCG/CCGTCG	1				1	0.018%
ACGGGC/CCCGTG	1				1	0.018%
ACTCCC/AGTGGG	1				1	0.018%
ACTCCT/AGGAGT	1				1	0.018%
ACTCGG/AGTCCG		2			2	0.036%
ACTCTC/AGAGTG	2	2			4	0.072%
ACTCTG/AGAGTC	2				2	0.036%
ACTGAT/AGTATC	1				1	0.018%
ACTGCC/AGTGGC	2		1		3	0.054%
ACTGCT/AGCAGT	1	1			2	0.036%
ACTGGG/AGTCCC	1				1	0.018%
AGAGAT/ATCTCT	3				3	0.054%
AGAGCC/CTCTGG	2				2	0.036%
AGAGCT/AGCTCT	1				1	0.018%
AGAGGC/CCTCTG	1	1		1	3	0.054%
AGAGGG/CCCTCT	8	4			12	0.216%
AGATGG/ATCTCC	2	6		1	9	0.162%
AGCAGG/CCTGCT	1			1	2	0.036%
AGCATG/ATGCTC		1			1	0.018%
AGCCCC/CTGGGG	1				1	0.018%
AGCCGC/CGGCTG		1			1	0.018%
AGCCTC/AGGCTG	1				1	0.018%
AGCCTG/AGGCTC			2		2	0.036%
AGCTCC/AGCTGG		1		1	2	0.036%
AGGATG/ATCCTC	4	1			5	0.090%
AGGCCC/CCTGGG		1			1	0.018%

AGGCGG/CCGCCT	2				2	0.036%
AGGGAT/ATCCCT	1				1	0.018%
AGGGGC/CCCCTG	2				2	0.036%
ATCATG/ATCATG	1				1	0.018%
ATCCCC/ATGGGG	3				3	0.054%
ATCCCG/ATCGGG		1			1	0.018%
ATCCGC/ATGCGG				1	1	0.018%

Supplementary Table 6

Characteristic features of the 29 novel EST-SSR markers in *S. incisa*

Primer	FORWARD PRIMER1 (5'-3')	REVERSE PRIMER1 (5'-3')	Tm(°C)	SSR	*Na*	*Ne*	*He*	*I*	*PIC*	*Fst*	*Nm*
S1	CTCTCCTTCACACTAGCTCGG	AACATGGCCTCGTACACACA	59.2	(TC)7	2	1.402	0.289	0.461	0.245	0.0051	48.4
S2	GATAGAGCGCAAAGTGGAGG	TTATGGCTCTCTCTCCCCAA	59.9	(GTG)6	2	1.750	0.432	0.620	0.336	0.0041	60.5
S3	TGATGACGTGCTTGTTCTCC	CACTCCCGGGAATCAAAAA	59.8	(GA)8	2	1.130	0.116	0.230	0.108	0.0005	464.5
S4	GCACTCAGGAGGGAGTGAAG	GGACCTGGACTTGGAGTTGA	59.9	(GAG)6	2	1.075	0.070	0.156	0.067	0.0067	37.2
S5	GCGAGAAAATAGTGTTAGTGTGAGA	CCGCTTTTACCCTTTGATGA	59	(GA)11	5	2.582	0.619	1.119	0.541	0.0581	4.0
S6	ACAGTCGACCCAGCATTACC	AGCAACTGAAACCCACCATC	59.9	(CCA)5	3	1.280	0.221	0.424	0.201	0.0031	80.5
S7	TCTCAAATCACTTCCGGACC	ATCAGACGGCAACAGGAGAC	60	(TCCCAT)4	3	2.761	0.643	1.051	0.560	0.0051	48.7
S8	AACAAACTGACCCCAAAACG	CCCCACCAAGAATTTTCAGA	59.8	(TTTC)4	4	3.569	0.726	1.324	0.669	0.0057	43.9
S9	TCCAGAGTCTTCCATTTGGG	CCCACCAAATAGCCAACAAC	60	(GTG)6	5	1.923	0.484	0.917	0.440	0.0036	69.7
S10	GAAACCCCCTTCCTTACCAA	GAGGGCCATGAAGTTGAGAA	60.1	(CT)8	2	1.965	0.496	0.684	0.370	0.0292	8.3
S11	TCGTAAAGCATGTCGTCGTC	GGAAGCACAGCAAGACATGA	59.8	(GAA)7	5	2.836	0.653	1.238	0.602	0.0034	73.2
S12	GATCATGAGAGACCCGGAAA	AGCAATTTAACGGCGACATC	60	(CCA)7	2	1.543	0.355	0.536	0.290	0.0095	26.0
S13	CATGTCAGAGAGGGGTCTCC	CAGAGGTTCGGAAGAGCATC	59.6	(CAC)5	2	1.407	0.291	0.464	0.247	0.0005	519.5
S14	GGCTTTGAAAATCCGATGAA	AGCCAGAGACACAAAAACCC	60	(AG)7	2	1.421	0.299	0.473	0.252	0.0001	1000.0
S15	CTGGCTTTTCAAGCACAGTCT	ATTTTTGCTTTGGGTTGTGG	59.6	(CT)10	3	2.692	0.634	1.042	0.555	0.0043	58.0
S16	CCAACGGTTCTAAACTCCCA	CAGAATTTGCAGAGCCTTCAG	59.9	(TC)7	3	1.805	0.450	0.792	0.404	0.0055	45.1
S17	TGGCTGACCAGTTCCTTCTT	AAATCCAACCCAACCCTACC	59.8	(GA)11	5	2.377	0.584	1.043	0.510	0.0019	133.7
S18	TTGTGGAGCACTTAGCTCGAT	TGAAGACGATGATGATGGGA	60	(TC)7	4	3.532	0.723	1.320	0.665	0.0276	8.8
S29	CCTACGAGCGTCTCTTGACC	ATTGATAACCCACCCATCCA	60.	(CAG)7	2	1.324	0.247	0.410	0.214	0.0054	45.8
S20	CTGTCTCAGCTTCCAGCCTT	GGCTAAGACCACCACTTGGA	59.7	(AC)7(TC)11	3	2.033	0.512	0.779	0.403	0.0073	34.0
S21	AAGATCCACAGCCTTCAGGA	TCATCATCACCACCATCACC	59.8	(TGA)5	6	2.933	0.665	1.210	0.592	0.0111	22.1
S22	GGGTTGTTCGAGGTCGTTTA	CAACGCTAACTTCACCAGCA	59.9	(TGG)5	2	1.781	0.444	0.630	0.342	0.0078	31.7
S23	ATTTTCATTTCCGTTCACGC	GGCCATGTCTAGTGGGTCTG	59.9	(TTAA)4	4	2.901	0.661	1.159	0.587	0.0259	9.4
S24	GTGGCGGTCGTAATCTGTTT	CGTCTCCTCTTTCTGTTGCC	60	(GCG)5	2	1.576	0.369	0.551	0.298	0.000	1000.0
S25	ACCCACATGGAATCTGGAAG	TCCTTGTGACATGTGTTGGAA	59.7	(GT)6	2	1.460	0.318	0.494	0.265	0.0103	24.0
S26	CTGGCTTTTCAAGCACAGTCT	ATTTTTGCTTTGGGTTGTGG	59.6	(CT)10	4	1.561	0.362	0.610	0.306	0.0368	6.5
S27	GCCTCTATTGCCATGTTGGT	ATCATCCAGTGGCTGGAAAG	59.9	(ACC)5	3	1.957	0.493	0.849	0.438	0.0037	66.4
S28	TTAACTGGTGGTTGCCCTTC	GGCTCTTCTATTGGGATGGA	59.9	(TCA)5	2	1.585	0.373	0.556	0.301	0.0102	24.2
S29	CATCGTCCTCCAATTCGTTT	GCTTCTCCAATTTCCCCTTC	59.9	(TCG)9	3	1.912	0.482	0.712	0.373	0.0010	245.5
mean					3	1.969	0.434	0.728	0.406	0.0117	21.1
min						1.130	0.070	0.230	0.108	0.0001	4.0
max						3.569	0.723	1.324	0.669	0.0368	519.5
